# Bioengineered Skin Grafts from Patient‐Derived Decellularized Extracellular Matrix and Autologous Cells for Personalized Regenerative

**DOI:** 10.1002/advs.202511889

**Published:** 2025-09-25

**Authors:** RaeHui Kang, Suyeon Shin, Yurim Choi, WonJun Jang, Soojin Park, Mi Kyung Lee, Hyun‐Jong Cho, Yu Shrike Zhang, Han‐Jun Kim, Bo Young Park, Junmin Lee

**Affiliations:** ^1^ Division of Interdisciplinary Bioscience & Bioengineering Pohang University of Science and Technology (POSTECH) Pohang 37666 Republic of Korea; ^2^ Department of Materials Science and Engineering Pohang University of Science and Technology (POSTECH) Pohang 37666 Republic of Korea; ^3^ Interdisciplinary Major Program in Innovative Pharmaceutical Sciences Korea University Sejong 30019 Republic of Korea; ^4^ College of Pharmacy Korea University Sejong 30019 Republic of Korea; ^5^ Department of Plastic and Reconstructive Surgery School of Medicine Ewha Womans University Seoul 03760 Republic of Korea; ^6^ Department of Pharmacy College of Pharmacy Kangwon National University Chuncheon 24341 Republic of Korea; ^7^ Division of Engineering in Medicine Department of Medicine Brigham and Women's Hospital Harvard Medical School Cambridge MA 02139 USA; ^8^ Department of Plastic and Reconstructive Surgery College of Medicine, Korea University Seoul 02841 Republic of Korea

**Keywords:** bioink, bioprinting, patient‐derived decellularized extracellular matrix (pddECM), regenerative medicine, skin tissue

## Abstract

The skin, as the body's largest organ, plays vital protective and regulatory roles, making it a key target in regenerative medicine. However, current skin models often lack patient specificity and fail to recapitulate native extracellular matrix (ECM) composition, limiting their clinical relevance. This study presents a 3D‐bioprinted skin model using a patient‐derived decellularized ECM (pddECM) bioink combined with keratin‐alginate (KA) bioink, mimicking native skin architecture and function. The pddECM supports high viability of human dermal fibroblasts (HDFs), promoting collagen I production and robust ECM remodeling, while the KA bioink enhances basal keratinocyte activation and cornification. The construct exhibits improved cell migration and angiogenesis, contributing to effective tissue integration and reduced hypoxic stress. Cytokine profiling reveals upregulation of ICAM‐1 and complement C5, which are associated with enhanced keratinocyte motility and rapid matrix remodeling, while downregulation of pro‐inflammatory cytokines (IL‐4 and IL‐8) suggests a favorable, fibrosis‐suppressive environment. In vivo, GelMA and GelMA+pddECM scaffolds accelerated wound closure without local or systemic toxicity, preserving dermal thickness and inducing migrating epidermal tongue (MET) expression. This patient‐specific, bioactive skin model holds strong potential as a next‐generation platform for personalized wound healing, drug screening, and high‐fidelity skin grafting in translational tissue engineering.

## Graphical abstract

This study presents a patient‐specific skin model using autologous cells, decellularized dermal ECM, and keratin–alginate bioinks. The model supports cell compatibility, collagen deposition, and epidermal differentiation within a biomimetic ECM environment.  It enhances angiogenesis while reducing pro‐fibrotic cytokines, creating a regenerative niche. In vivo results confirm its biocompatibility and wound healing potential, demonstrating its promise for personalized skin substitutes in regenerative medicine.

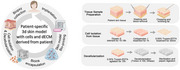

## 1 Introduction

The skin, the largest organ of the body, protects the body from external factors such as UV radiation, harmful chemicals, and pathogens, while maintaining homeostasis.^[^ ^1^ ^]^ It is composed of three layers: the epidermis, dermis, and hypodermis, along with its appendages.^[^ ^2^ ^]^ Skin complications resulting from burns or inflammation are typically treated with autologous skin grafts or acellular dermal matrices.^[^ ^3^ ^]^ Among these, the most effective methods are autologous skin grafting and autologous cell‐based therapies. These approaches offer benefits such as reduced risk of immune rejection, personalized treatment, enhanced tissue integration, and accelerated wound healing.^[^ ^4^ ^]^ Notably, autologous cells can be efficiently harvested in large quantities from a small amount of tissue.^[^ ^5^, ^6^ ^]^ However, autologous skin grafts remain limited by donor site availability and scarring, which may lead to functional impairments.

In clinical practice, there has been a continuous demand for skin substitutes that eliminate the need for donor site morbidity. To address this demand, recent advances in 3D bioprinting have enabled the creation of skin constructs that mimic the structure and function of native skin. This technique involves additive manufacturing using bioinks composed of living cells, biomaterials, and bioactive factors, precisely deposited in the desired configuration. Models incorporating keratinocytes, fibroblasts, and human umbilical vein endothelial cells (HUVEC), as well as those replicating appendages and microstructures, have been developed.^[^ ^7^ ^]^ Previous studies have focused on structural mimicry, with limited integration of tissue‐specific components and limited patient‐specific applicability.

Decellularized tissue‐derived bioinks have been developed to precisely replicate the extracellular matrix (ECM) by retaining essential components, such as collagen and glycosaminoglycans (GAGs), while eliminating immunogenic materials. Decellularized extracellular matrix (dECM) promotes tissue‐specific effects and facilitates cell growth and wound healing through its retained bioactive molecules and fibrous microstructure.^[^ ^8^ ^]^ Porcine skin‐derived dECM bioinks reduce contraction and enhance ECM remodeling, promoting wound closure and neovascularization.^[^ ^9^ ^]^ The fibroblast‐derived dECM models promoted wound contraction and angiogenesis, suggesting the formation of appendages and microstructures such as hair follicles and the subepidermal nerve plexus.^[^ ^10^ ^]^ Furthermore, the decellularized adipose tissue promoted anti‐inflammatory responses, re‐epithelialization, collagen accumulation, alignment, and angiogenesis.^[^ ^11^ ^]^ Notably, dECM models from tissues like the heart, lungs, and uterus have demonstrated the potential to enhance tissue‐specific functions and expressions, supporting reprogramming, regeneration, and increased native marker expression.^[^ ^3^, ^12^, ^13^ ^]^

**Scheme 1:**
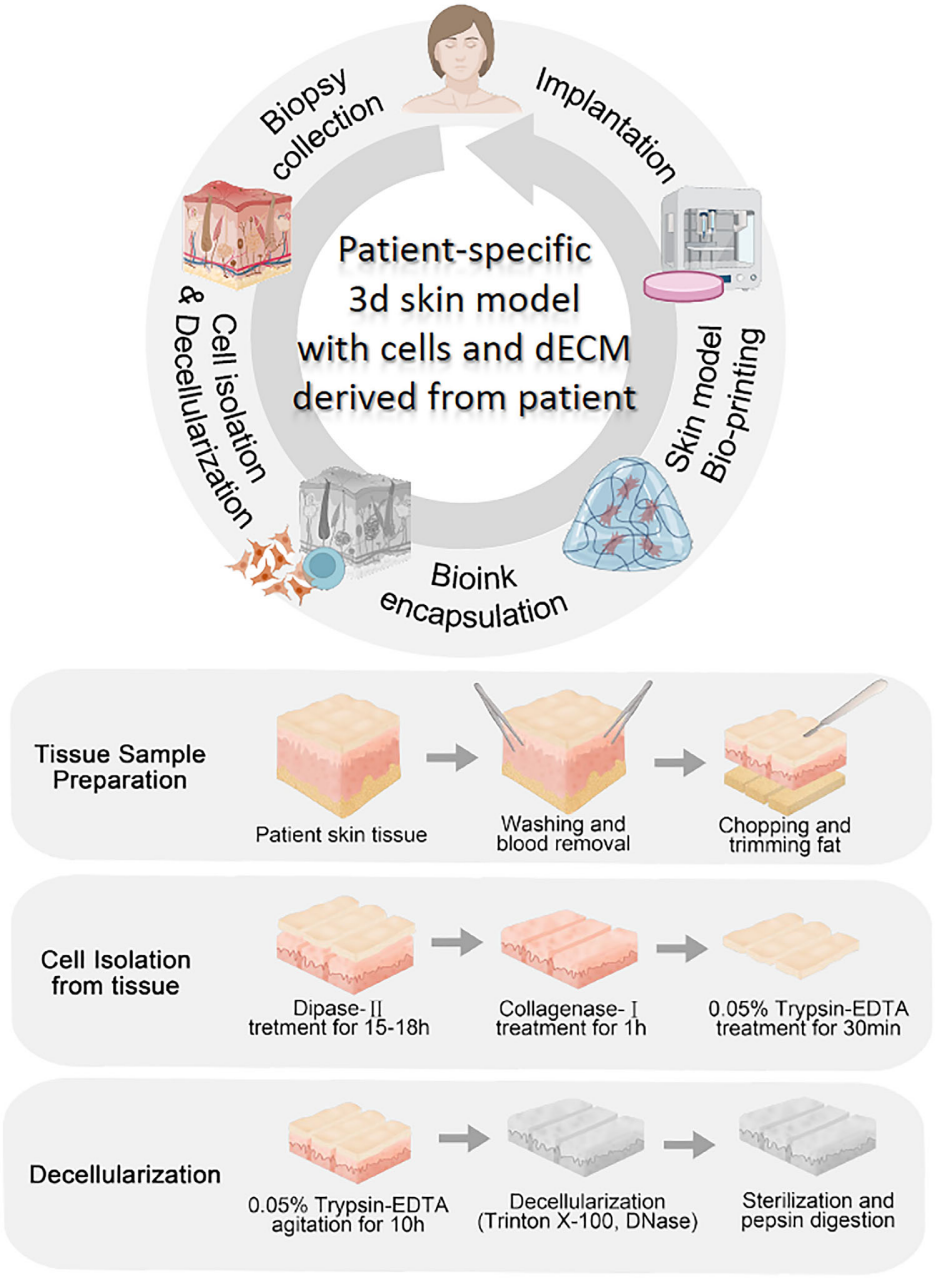
Graphical abstract of patient‐specific 3D skin model incorporating pddECM and autologous cells.

Natural and synthetic polymers offer tunable mechanical properties and printability but lack biological complexity. In contrast, dECM provides tissue‐specific bioactivity and native microarchitecture, enabling precise in vivo mimicry. This study integrates the complementary advantages of both materials to develop a patient‐specific skin model with both mechanical stability and biological functionality (**Scheme** **1**). To begin, fibroblasts and keratinocytes were isolated from patient skin tissues using dispase II, collagenase I, and trypsin‐ethylenediaminetetraacetic acid (EDTA). The dermal tissues were then decellularized to create pddECM bioink, alongside an epidermal bioink based on a keratin‐alginate (KA). To enhance the mechanical integrity and printability of the bioinks, gelatin methacryloyl (GelMA) and alginate were incorporated into both formulations. Using these two bioink components, we developed a patient‐specific 3D skin model. The pddECM retained proteins identical to those in the native tissue, with ECM remodeling processes predominating in biological activity. In the constructed model, the expression of epidermis‐specific markers, including Keratin 10 (KRT10), Keratin 14 (KRT14), and filaggrin (FLG), was analyzed. Collagen I synthesis and deposition, as well as cell migration within the dermal layer, were also evaluated. Cytokine assays were conducted to assess the potential effects of pddECM on wound healing, as it would occur in vivo. Subsequently, in vivo study showed that GelMA and GelMA+pddECM did not exhibit significant local or systemic toxicity, and accelerated skin wound healing by increasing migrating epidermal tongue (MET) and maintaining dermal thickness.

## 2 Results and Discussion

### 2.1 Isolation and Characterization of Autologous Dermal Fibroblasts and Epidermal Keratinocytes

**Figure 1:**
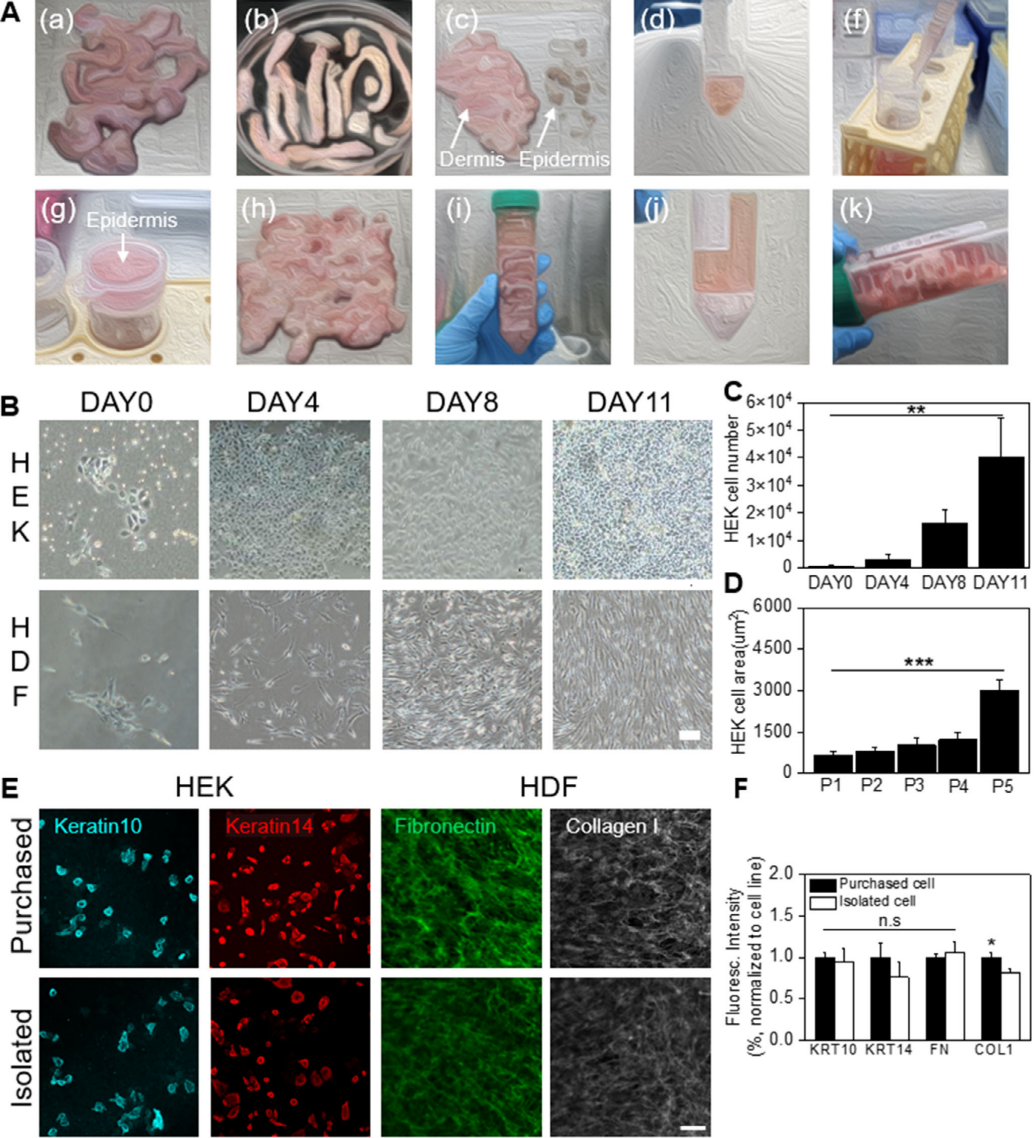
Isolation of cells from patient skin tissue. A) Isolation of patient‐derived fibroblasts and keratinocytes from patient tissue. B) Optical microscopy images of keratinocyte and fibroblast morphology observed. C) Time‐dependent cell count data showing the proliferation of keratinocytes (*n* = 3, ^**^ *p* < 0.005). D) Time‐dependent cell area showing the proliferation of keratinocytes (*n* = 3, ^***^ *p* < 0.0005). E) Immunofluorescence staining for keratin 14, keratin 10, fibronectin, and collagen type I in keratinocytes (HEK) and dermal fibroblasts (HDF) derived from patient skin tissue (“Isolated”) and commercially obtained primary cells (“Purchased”). F) Quantification of fluorescence intensity of the immunofluorescence staining shown in (E). Marker expression levels in patient‐derived cells were normalized to those in purchased cells (*n* = 4, ^*^ *p* < 0.05, ns: no significance).

Tissue regeneration using autologous cells accelerated healing by promoting the migration of host cells to the damaged area and triggering the early formation of new epidermal layers.^[^ ^14^, ^15^ ^]^ Autologous dermal fibroblast and epidermal keratinocyte therapies, such as CureSkin inj. (S.BIOMEDICS), Holoderm (TEGO SCIENCE), and ReNovaCell (Avita Medical Ltd.) have been widely used in skin repair and regeneration. To effectively utilize the regenerative potential of these therapies, we followed a standardized protocol to isolate autologous dermal fibroblasts and keratinocytes from the skin tissue, ensuring both cell viability and functionality for therapeutic use. The following procedures were performed to isolate keratinocytes and fibroblasts from patient skin tissue stored at 4 °C: 1) Tissue samples were washed three times with 1× phosphate‐buffered saline (PBS) to remove coagulated blood and impurities. The adipose layer, hair, and dissociated tissues were also removed. 2) The tissue was cut into pieces with a width of 2–3 mm. With the epidermal layer facing up, the biopsy was treated with dispase II solution and was incubated at 4 °C for 15–18 h. 3) The epidermis and dermis were separated using forceps, followed by a PBS wash to remove any remaining dispase II solution. 4) The epidermis was incubated at 37 °C with 0.05% trypsin‐EDTA for 30 min with shaking. 5) By adding the culture medium, the trypsin reaction was stopped, and gentle pipetting was performed to isolate the cells from tissue. 6) The epidermal tissue was filtered using a cell strainer, and the remaining tissue was vortexed with fresh cell culture media and filtered again. 7) The isolated dermis was finely minced into small fragments. 8) Th dermal tissue was treated with collagenase‐1 solution at 37 °C for 30–60 min. 9) HDF were dissociated from the tissue by pipetting, and the collagenase solution was removed via centrifugation. 10) The process of adding fresh cell culture media and centrifuging to remove the collagenase solution was repeated three times. Finally, the cells were resuspended in fresh media and seeded into a flask (**Figure** **1**A).

Fibroblast‐derived dermis exhibited a spindle‐shaped morphology. As the passage number increased, the cells became longer and wider.^[^ ^16^, ^17^ ^]^ As aging progressed, the nuclei became enlarged and hypertrophic; the keratinocytes displayed a cobblestone‐like arrangement, and as they proliferated, the cells became more closely connected, and as the cells underwent differentiation, the cytoplasm expanded, and the cells became flatter.^[^ ^18^ ^]^ Similarly, HEK and HDF isolated from the skin tissue also exhibited such characteristic morphology (Figure 1B), where the proliferation rate of HEK was increased by 51.64‐fold on day 11 compared to day 0 (Figure 1C). The area of HEK remained stable for up to passage 4 (1.88‐fold change compared to passage 1), followed by a marked increase at passage 5 (4.62‐fold change compare to passage 1) (Figure 1D). Verification of the isolated cells as HEK and HDF was performed using immunofluorescence staining for KRT10, KRT14, collagen type I, and fibronectin (FN). Marker expression profiles were compared between commercially obtained primary cells and patient‐derived cells (Figure 1E). No significant differences were observed in the expression of KRT10, KRT14, and FN; however, collagen type I expression showed a slight decrease (≈0.87‐fold compared to purchased HDF) (Figure 1F). This reduction is considered within the range of biological variability, potentially resulting from differences in cell origin, donor‐specific characteristics of the patient‐derived cells, and variations in cellular state under short‐term culture conditions. During keratinocyte culture, a small number of melanocytes were occasionally present, which were removed using 0.05% trypsin‐EDTA treatment at room temperature, following established protocols.^[^ ^19^ ^]^ The presence of melanocytes in keratinocyte culture suggested that skin homeostasis is being maintained and that proper cell‐cell interactions were occurring.^[^ ^20^ ^]^

### 2.2 Development and Characterization of Patient‐Derived Decellularized Dermal ECM Bioink

To formulate the bioink that mimics native dermal tissue, a decellularization process was selected. Native tissue consists of a complex microstructure, including fibrous proteins such as collagen, FN, and elastin, along with other essential components such as glycosaminoglycans, glycoproteins, proteoglycans, and growth factors. These components play a crucial role in promoting tissue regeneration and repair through physical and chemical interactions with cells, regulating processes such as cell adhesion, growth, migration, and differentiation.^[^ ^21^ ^]^ Many researchers have developed tissue engineering models using natural biomaterials (collagen‐, fibrinogen‐, and elastin‐based matrices) and synthetic polymers (GelMA and hyaluronic acid) to mimic these components for skin grafts.^[^ ^22^, ^23^, ^24^ ^]^ However, skin models based on natural and synthetic polymers are deficient in components such as fibrous proteins, glycoproteins, and polysaccharides compared to native. To address these limitations, research into dECM has advanced. This approach demonstrates improvements in cellular activity, tissue‐specific functions, and gene expression, providing a more biomimetic environment compared to natural or synthetic polymer models.^[^ ^25^, ^26^, ^27^ ^]^ Consequently, a bioink rich in these critical components was developed, closely mimicking the native dermal ECM to better support cellular functions and enhance tissue‐specific outcomes.

**Figure 2:**
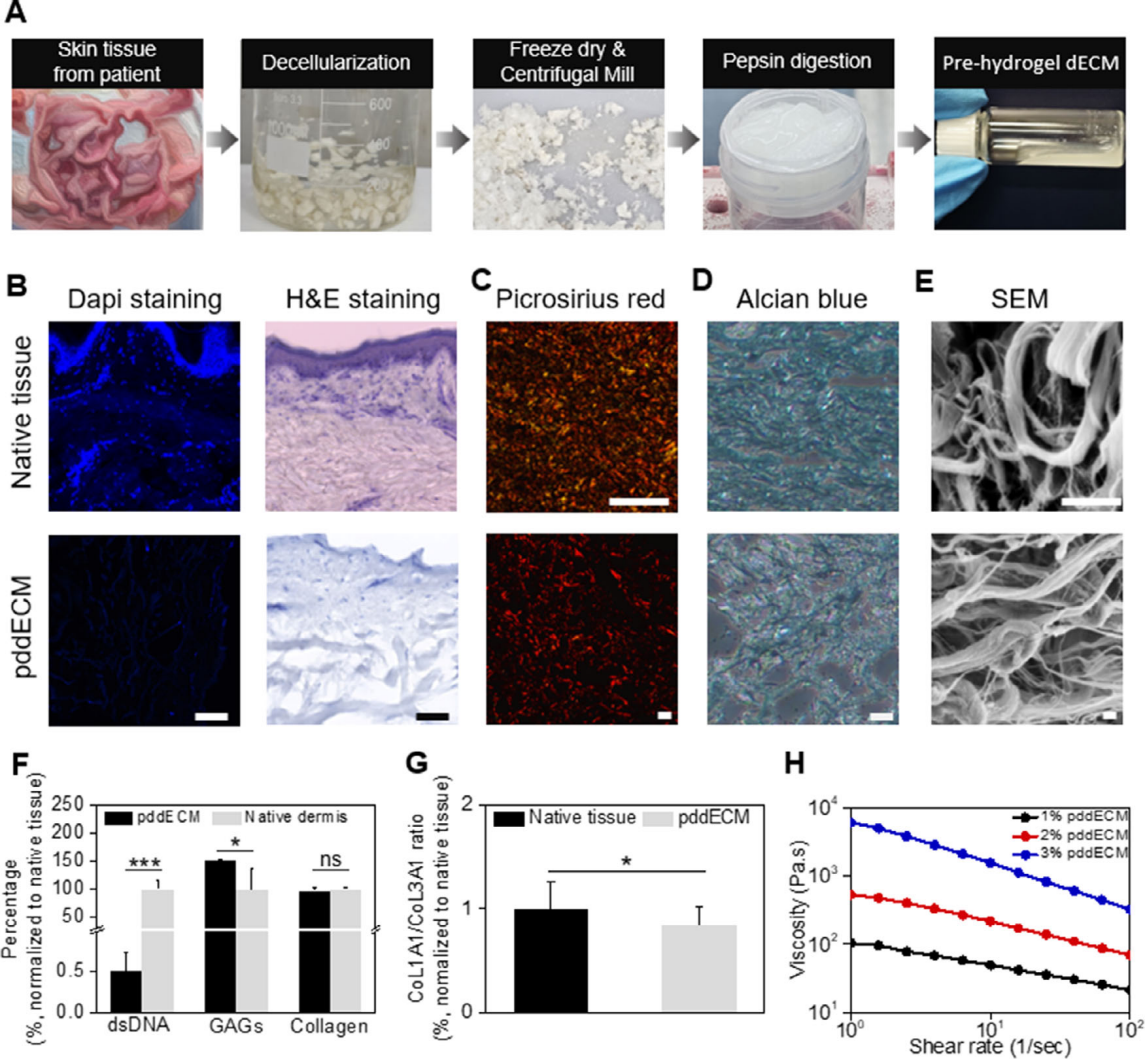
Preparation of characterization of human skin tissue‐derived dECM. A) Representative images of the five‐step process from decellularization to pre‐hydrogel preparation. B) DAPI and H&E staining before and after decellularization process. C) Picrosirius red staining before and after decellularization process. D) Representative images of alcian blue staining to verify GAG preservation. E) SEM images for pddECM and native tissue. F) Analyses of residual dsDNA contents and ECM components (GAGs and collagen) in the pddECM compared to native dermis tissue (*n* = 3, ^***^ *p* < 0.0005, ^*^ *p* < 0.05, ns: no significance). G) Quantification of immunofluorescence intensity of collagen 1 alpha 1/collagen 3 alpha 1 ratio (*n* = 3, ^*^ *p* < 0.05). H) Shear rate‐viscosity profile at different pddECM concentrations. Scale bars = 100 µm.

To obtain dECM from patient‐derived dermal tissue, a decellularization process involving chemical reagents (Trypsin‐EDTA, Triton X‐100, DNase, and MgCl_2_) in conjunction with physical methods such as agitation and cutting was employed (**Figure** **2**A). Hematoxylin and eosin (H&E) staining and 4′,6‐diamidino‐2‐phenylindole (DAPI) staining confirmed the effective removal of immunogenic components from pddECM (Figure 2B). Collagen preservation was confirmed via Picrosirius staining (Figure 2C), and preservation of GAGs was verified with Alcian blue staining (Figure 2D). Scanning electron microscopy (SEM) further demonstrated the preservation of the complex microstructure, highlighting the retention of the intricate porous architecture, comprising both thin and thick fibers (Figure 2E). In addition, DNA quantification showed a significant reduction in DNA content (≈0.01‐fold compared to native tissue), while levels of GAGs and collagen exhibited minimal alterations (≈1.51‐ and ≈0.90‐fold, respectively, compared to native tissue) (Figure 2F; Figure S1, Supporting Information). The DNA concentration fell below the FDA‐approved threshold of 50 ng (Figure S2, Supporting Information). This confirmed that the decellularization process was effectively executed, indicating a reduction in potential immune and inflammatory responses.^[^ ^28^ ^]^ Additionally, picrosirius red staining and immunofluorescence staining were conducted to evaluate the collagen composition in native dermal skin and pddECM.^[^ ^29^ ^]^ While slight changes in collagen proportions were observed after decellularization (≈0.85‐fold compared to native tissue), these differences were not statistically significant (*p* = 0.8742) (Figure 2G). Moreover, rheological analysis of pddECM across different concentrations confirmed its shear‐thinning properties, underscoring its potential application as a bioink (Figure 2H).

### 2.3 Proteomic Analysis and Functional Preservation of Patient‐Derived dECM

**Figure 3:**
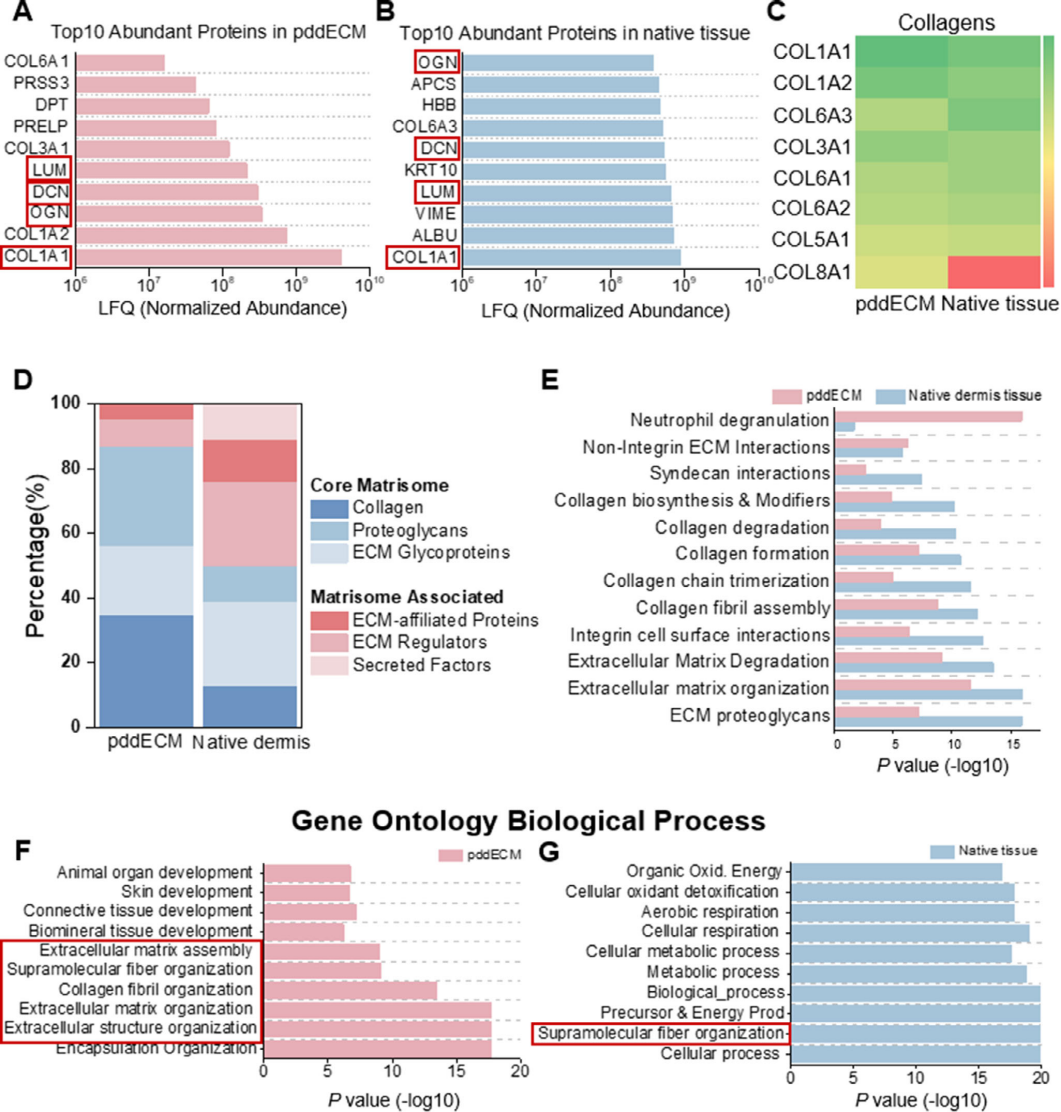
Proteomic analyses of pddECM and native dermis tissue. A) Top ten abundant proteins in pddECM. B) Top ten abundant proteins in native tissue. C) Heatmap of collagen in pddECM and native tissue. D) Matrisome profile of pddECM and native tissue. E) Reactome analyses and F) GO‐BP terms overrepresentation analysis in pddECM. G) GO‐BP terms overrepresentation analyses in native tissue.

To verify whether the protein composition of the pddECM is preserved similarly to that of native dermis tissue, a protein profile analysis was performed for both pddECM and native dermis tissue. Analysis of the top 10 common proteins in each sample revealed that collagen type I alpha 1 (COL1A1), osteoglycin (OGN), lumican (LUM), and decorin (DCN) were shared. The most abundant proteins identified in pddECM included COL1A1, collagen type I alpha 2 (COL1A2), collagen type III alpha 1 (COL3A1), collagen type VI alpha 1 (COL6A1), osteoglycin (OGN), decorin (DCN), lumican (LUM), dermatopontin (DPT), protease serine 3 (PRSS3), and proline and arginine‐rich end leucine‐rich repeat protein (PRELP) (**Figure** **3**A). In native dermis tissue, the top 10 most abundant proteins were identified as COL1A1, collagen type VI alpha 3 (COL6A3), OGN, DCN, LUM, albumin (ALBU), KRT10, hemoglobin subunit beta (HBB), and serum amyloid P component (APCS) (Figure 3B). DCN found in pddECM plays a crucial role in collagen fibrillogenesis and interacts with growth factors such as TGF‐β, which are important for preventing fibrosis.^[^ ^30^, ^31^ ^]^ LUM is involved in collagen fibril formation, regulating collagen deposition in the skin, and maintaining skin integrity during wound healing.^[^ ^32^ ^]^ In addition, OGN and DPT influence collagen fibril formation and alignment, promoting cell adhesion and matrix assembly, thereby contributing to wound healing.^[^ ^33^, ^34^, ^35^ ^]^ PRELP stabilizes the interactions between the ECM and collagen fibrils, preventing excessive collagen degradation.^[^ ^36^, ^37^ ^]^

In native skin, various types of collagen form a complex network. Collagen type I constitutes 80–85%, type III accounts for 8–11%, with small amounts of collagen types V and VI also present.^[^ ^7^, ^8^, ^38^, ^39^ ^]^ A heatmap analysis was conducted to compare the distribution of different collagen types before and after decellularization and to evaluate the preservation of collagen composition (Figure 3C). In pddECM, collagen type I (COL1A1 and COL1A2) and collagen type III (COL3A1) showed a slight increase (≈1.074, ≈1.076, and ≈1.046‐fold changes compared to native dermis tissue), while collagen type VI (COL6A1, COL6A2, and COL6A3) remained relatively stable (≈0.933, ≈0.956, and ≈0.829‐fold changes compared to native dermis tissue).

The percentages of core matrisome and matrisome‐associated proteins were calculated to compare the compositional differences between pddECM and native tissue, aiming to enhance understanding of the structural and functional variations in the ECM (Figure 3D). The collagen, proteoglycans, and ECM glycoproteins in pddECM accounted for ≈34.78%, 30.43%, and 21.74%, respectively (≈2.67‐, ≈2.80‐, and ≈0.83‐fold changes compared to native tissue). However, the percentages of ECM regulators and ECM‐affiliated proteins decreased (≈0.33‐ and ≈0.33‐fold changes compared to native tissue), with no secreted factors detected. Reactome analysis confirmed that the decellularized ECM retains key biological pathways of native dermal tissue to a considerable extent (Figure 3E). Essential pathways such as ECM proteoglycans, ECM organization, ECM degradation, integrin cell surface interactions, and collagen fibril assembly were preserved even after decellularization (Figure 3F & 3G). This suggests that the pddECM could replicate a complex protein network, potentially reproducing the structural and physiological functions of the original tissue to a certain extent.

### 2.4 Optimization of Mechanical and Printability Properties of dECM‐Based Bioinks

**Figure 4:**
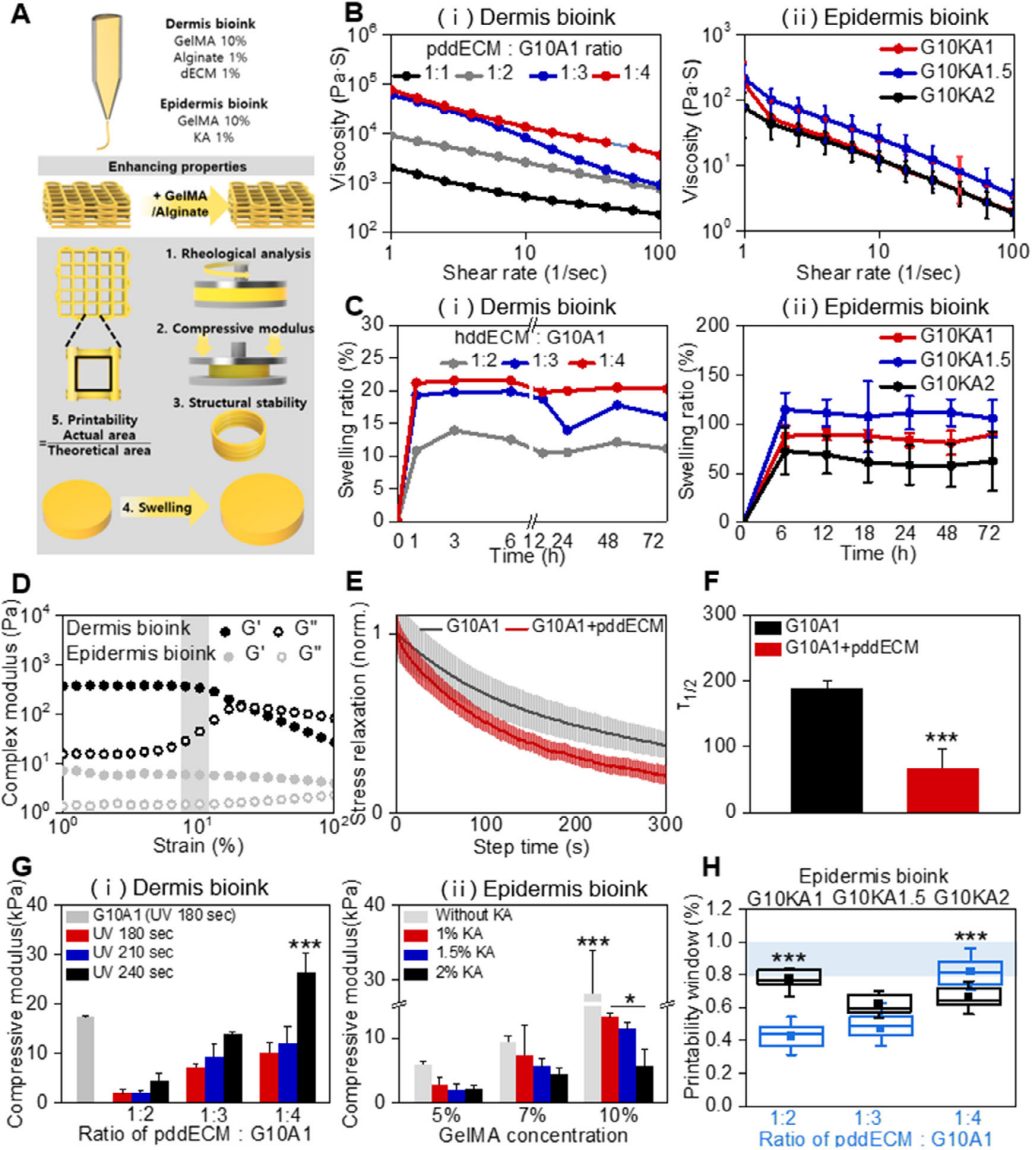
Material characterizations of the bioinks: mechanical, rheological, and printability properties. A) Illustration of the bioink composition and the experimental procedures conducted to evaluate rheological properties, compressive modulus, structural stability, swelling behavior, and printability. B) Shear rate‐viscosity profiles in dermis bioink and epidermis bioink. C) Swelling ratio analyses of the bioink, demonstrating the extent of swelling over time. D) Complex moduli of dermal and epidermal bioinks depending on the changes in strain. E) Stress relaxation of dermal bioinks (G10A1 ± pddECM) at 10% strain, normalized to initial stress (step time = 0 s). F) Half relaxation time (T_1_/_2_) from (E), showing faster stress dissipation with pddECM (*n* = 3, ^***^ *p* < 0.0005). G) Compressive moduli of dermal and epidermal bioink hydrogels in different conditions (*n* = 3, ^***^ *p* < 0.0005, ^*^ *p* < 0.05). H) Printability analyses of dermal‐ and epidermal bioink based on its composition (*n* = 3, ^***^ *p* < 0.0005).

Since pddECM is solubilized, it exhibits limitations including low mechanical strength, reduced print fidelity, limited printability, and high susceptibility to degradation (**Figure** **4**A). To address these limitations, dECM was formulated into an ink by blending it with synthetic polymers or biopolymers.^[^ ^9^, ^40^, ^41^, ^42^, ^43^ ^]^ All conditions demonstrated the shear‐thinning behavior, characterized by a decrease in viscosity with increasing shear rate (Figure 4B). A swelling test was conducted to assess behavior of the skin model upon exposure to physiological fluids. For the dermal layer bioink, swelling began within 1 h. As the GelMA content was increased, the hydrogel exhibited swelling ratios of 10.83%, 19.29%, and 21.20%, respectively. In contrast, the epidermal layer bioink swelled over a period of 6 h, with swelling sustained up to 72 h (≈1.02, 0.92, and 0.86‐fold changes relative to the 72‐h value) (Figure 4C). The swollen hydrogel is expected to function as a hemostatic agent by applying localized pressure after adhesion to the skin.^[^ ^44^ ^]^ The storage modulus (G′) and loss modulus (G″) of each dermal and epidermal layer hydrogel were measured as a function of strain. For both bioinks, the storage modulus exceeded the loss modulus within the low strain range (0.1–10%), indicating a predominantly elastic behavior (G′ > G″) (Figure 4D). It was confirmed that the gel state was maintained even at 10% strain, which represents the maximum strain typically encountered in physiological conditions.^[^ ^45^ ^]^ The frequency‐dependent complex modulus and stress relaxation behavior of the extracellular matrix are known to influence cell fate by regulating nuclear architecture, chromatin reorganization, and gene accessibility.^[^ ^46^, ^47^ ^]^ In this study, the complex modulus and stress relaxation behavior were measured under a 10% strain condition. In the frequency sweep (0.1–10 Hz), both conditions exhibited elastic dominance at low frequencies (tan δ < 1), while G10A1+dECM1 displayed lower complex moduli (G′ and G″) across the entire frequency range compared to G10A1 (Supplement S3). In the stress relaxation test, G10A1+dECM1 showed a faster relaxation rate, with the half‐relaxation time (t_1_/_2_) reduced to ≈0.35‐fold relative to G10A1. Such rapid stress relaxation has been reported to promote mechanically mediated cellular processes, including cell spreading, proliferation, and differentiation (Figure 4E & 4F). The compressive modulus of the dermal ink hydrogel was evaluated based on varying UV cross‐linking times under a 1:4 ratio. After 180 s of cross‐linking, the modulus values were measured at 2.00, 7.19, and 10.16 kPa, respectively, showing a clear increase in modulus as the cross‐linking time was extended (≈2.20‐, 1.93‐, and 2.60‐fold changes between 180 and 240 s) (Figure 4G). In the case of the epidermal layer ink, the modulus decreased as KA bioink concentrations of 5%, 7%, and 10% were added to GelMA, resulting in reductions of ≈0.79‐, 0.61‐, and 0.43‐fold when comparing the KA bioink 1% and KA bioink 2% conditions (Figure 4H). Butenko et al. demonstrated that high‐modulus hydrogels induce fibrosis, scarring, and inflammation, while low‐modulus hydrogels enhance tissue integration, accelerate wound regeneration, and promote M2 macrophage polarization.^[^ ^48^ ^]^ Based on these findings, we optimized the bioink formulation through printability analysis, identifying the 1:4 ratio as optimal. This composition corresponded to a crosslinking time of 210 s with GelMA 10% and KA bioink 1% (Figure 4I). Print fidelity, evaluated as θ = theoretical area (S_0_) / actual area (S), showed average values of 0.833, 0.473, and 0.423 (± 0.096, 0.104, and 0.072, respectively) for 1:4, 1:3, and 1:2 ratios in the dermal layer ink, with only the 1:4 ratio falling within the suitable range (*p* < 0.0005 compared to 1:2 and 1:3; Figure 4I). To verify structural integrity, a hollow cylinder was printed; while 1:2 and 1:3 ratios collapsed or failed to stack, the 1:4 ratio achieved stable stacking.

### 2.5 Evaluation of Epidermal Differentiation and Collagen Synthesis in the Bioengineered Skin Model

**Figure 5:**
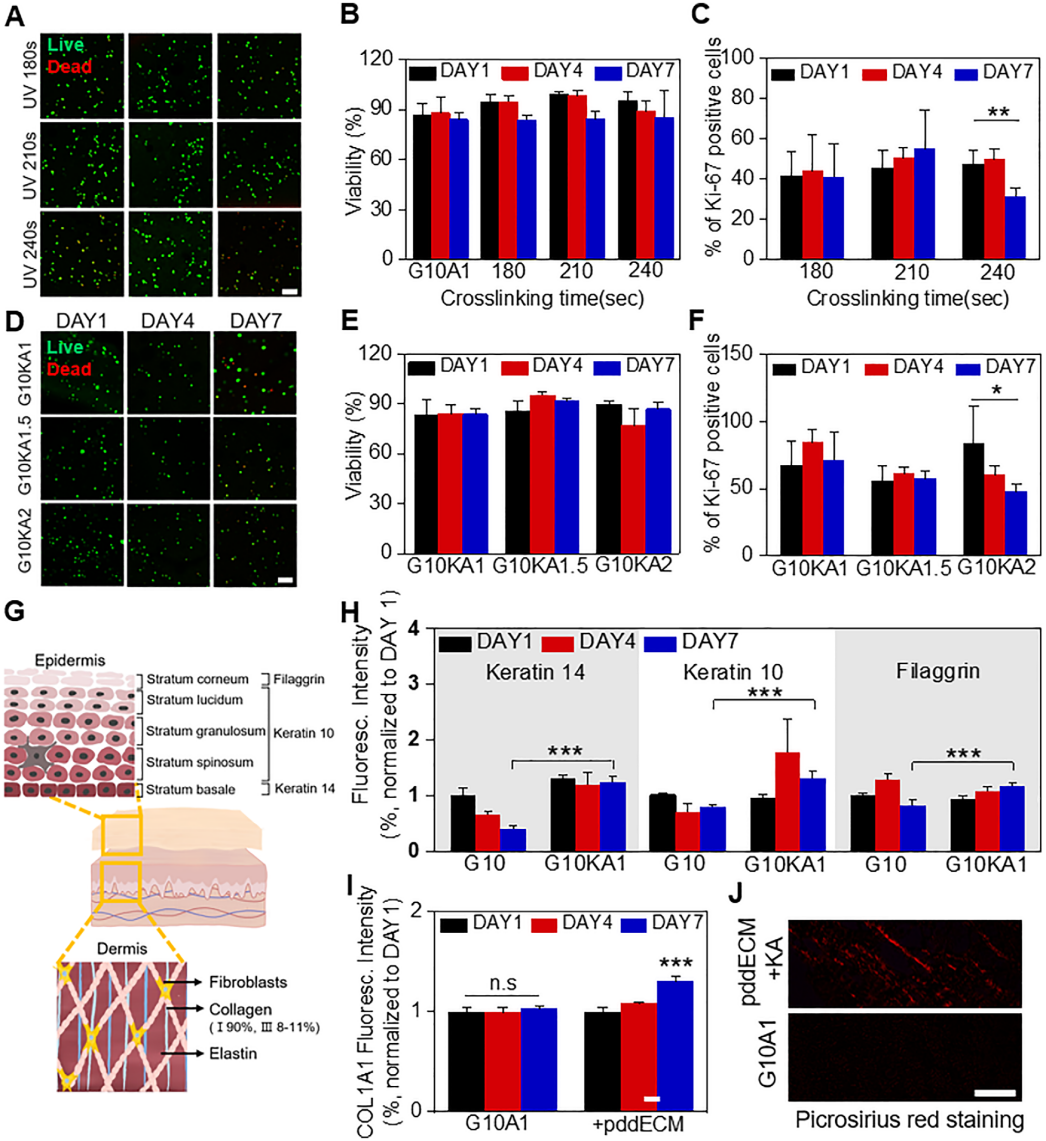
Assessment of cell viability, proliferation, and marker expressions in pddECM and KA bioinks. A) Representative immunofluorescence images of live/dead HDFs in dermal bioink. Calcein‐AM (live in green) and ethidium homodimer‐1 (dead in red). Quantitative B) viability and C) Ki‐67 positive cells of HDFs cultured in dermal bioinks for 1, 4, and 7 days (*n* = 3, ^**^ *p* < 0.005). D) Representative immunofluorescence images of live/dead HEK in epidermal bioink. Calcein‐AM (live in green) and ethidium homodimer‐1 (dead in red). Quantitative E) viability and F) Ki‐67 positive cells of HEKs cultured in epidermal bioinks for 1, 4, and 7 days (*n* = 3, ^*^ *p* < 0.05). G) Illustration of skin structure, including the epidermis and dermis, along with their respective components and expressions of markers. H) Quantification of immunofluorescence intensity for the expressions of markers KRT10, KRT14, and filaggrin, assessing epidermal function markers with and without KA ink (*n* = 5, ^***^ *p* < 0.0005). I) Normalized intensity of collagen 1 expression, evaluated through immunofluorescence staining (*n* = 5, ^***^ *p* < 0.0005, ns: no significance). J) Picrosirius red staining for ours model and control. Scale bar = 100 µm.

The pddECM was treated with various reagents during the decellularization process and digested with pepsin under acidic conditions. To assess the impact of residual pddECM components on cell viability and proliferation, live/dead staining and Ki‐67 staining were performed. The effect of incorporating KA into the epidermal bioink was evaluated as well. In the dermal layer, HDF viability on day 7 decreased by 11.23% (180 s), 15.29% (210 s), and 10.62% (240 s) compared to day 1. However, the HDF viability on day 7 remained high at 83.67% (± 2.59%, 180 s), 83.95% (± 4.67%, 210 s), and 85.32% (± 15.61%, 240 s), suggesting that a certain level of viability was maintained over time in the dermal layer (**Figure** 5A & 5B). Ki‐67 levels for the 180‐ and 210‐s conditions were maintained or slightly increased on day 7 (≈0.99‐fold and ≈1.21‐fold changes compared to day 1). However, for the 240‐s condition, the percentage of Ki‐67‐positive cells decreased on day 7 (≈0.66‐fold change compared to day 1) (Figure 5C; Figure S4, Supporting Information). In the epidermal layer, the HEK viability of the G10KA1, G10KA1.5, and G10KA2 (GelMA 10% & KA 1%, 1.5%, and 2%, respectively) conditions on day 7 was similar to that observed on day 1 (≈1.00‐, ≈1.06‐, and ≈0.97‐fold changes) (Figure 5D & 5E). Additionally, the percentage of Ki‐67‐positive cells in the G10KA1 and G10KA1.5 conditions was maintained on day 7 (≈1.06‐ and ≈1.03‐fold changes compared to day 1), whereas the G10KA2 condition showed a marked decrease (≈0.57‐fold change compared to day 1) (Figure 5F; Figure S5, Supporting Information).

The skin consists of two primary layers: the epidermis and dermis (Figure 5G). The epidermis is composed of various cell types, including keratinocytes, melanocytes, Langerhans cells, and Merkel cells, along with dECM components. KRT10, primarily expressed in the stratum granulosum and stratum spinosum, is involved in epidermal differentiation and structural maintenance. KRT14, found in the stratum basale, contributes to basal cell stability. FLG, localized in the stratum granulosum, plays a role in the uppermost stratum corneum.^[^ ^49^, ^50^, ^51^, ^52^, ^53^ ^]^ By analyzing KRT10, KRT14, and FLG markers, we assessed whether our model effectively maintains proper cellular processes and epidermal integrity. The dermis is composed of several key elements, including fibroblasts, collagen fibers, and elastin. Fibroblasts synthesize and remodel collagen and elastin fibers, providing skin with strength and elasticity. The collagen in the dermis is predominantly made up of collagen type I (≈80%) and collagen type III (≈15–20%). Collagen type I imparts tensile strength to the skin, while collagen type III provides flexibility, playing a crucial role in the skin's elasticity and in tissue repair during wound healing.^[^ ^54^, ^55^, ^56^ ^]^ Thus, we evaluated COL1A1 and COL3A1 to assess collagen synthesis capacity.

To investigate the effect of KA bioink on marker expressions, immunofluorescence staining was performed (Figure 5H; Figure S5, Supporting Information). Regarding the expression of KRT14, the G10 condition showed a reduction by day 7 (≈0.40‐fold change compared to day 1), whereas the G10KA1 condition exhibited a slight decrease but was generally maintained (≈0.94‐fold change compared to day 1). For KRT10 expression, the G10 condition demonstrated a modest reduction by day 7 (≈0.79‐fold change compared to day 1), while the G10KA1 condition showed a notable increase (≈1.34‐fold change compared to day 1). In terms of FLG expression on day 7, G10 showed a decrease (≈0.82‐fold change compared to day 1), while G10KA1 exhibited an increase (≈1.25‐fold change compared to day 1). Interestingly, in the G10 condition, FLG expression initially increased markedly on day 4 (≈1.28‐fold change compared to day 1) but then sharply declined by day 7 (≈0.64‐fold change compared to day 4). The decrease in FLG expression indicates shedding and desquamation of the stratum corneum.^[^ ^57^, ^58^ ^]^ In contrast, FLG expression in G10KA1 gradually increased (≈1.22‐fold change compared to day 1), indicating that the early stages of differentiation and the process of strengthening the skin barrier are in progress.^[^ ^59^, ^60^ ^]^

To evaluate intercellular tight junction formation, Zonula occludens‐1 (ZO‐1) immunofluorescence staining was performed on Day 14 (Figure S7, Supporting Information). Due to the low cell density used for epidermal printing (2 × 10⁶ cells mL^−1^), a fully stratified epidermis was not observed. However, transepithelial electrical resistance (TEER) measurements were additionally conducted over time to assess barrier formation (Figure S8, Supporting Information). TEER values were normalized to the G10 condition at Day 1; at Day 14, the G10 group exhibited a 2.26‐fold increase, and the G10KA1 group showed a 3.03‐fold increase relative to their respective Day 1 values, with the latter demonstrating a statistically significant elevation. These findings suggest that the rise in electrical resistance is more likely attributable to cell proliferation, spreading, increased coverage, and enhanced cell–matrix adhesion rather than the contribution of densely organized tight junctions.^[^ ^61^ ^]^ Collectively, these results indicate that, although the epidermis in our model did not achieve full keratinization, it retained partial functional barrier properties and holds promise as a graft material capable of infiltration, integration, and remodeling within host tissue after implantation.

To confirm collagen synthesis influenced by pddECM, the COL1A1/COL3A1 ratio was analyzed (Figure 5I; Figure S9, Supporting Information). The ink without pddECM showed a slight decrease in the ratio (≈0.72‐fold change from day 1 to 7). In contrast, the ink with pddECM exhibited a significant increase in the ratio (≈2.45‐fold change from day 1 to 7). When comparing the COL1A1/COL3A1 ratio on day 7 between the two ink conditions, the ink containing pddECM showed a 3.02‐fold higher ratio (*p* < 0.005). These findings suggest that the presence of pddECM significantly enhances collagen type I synthesis, as evidenced by the elevated COL1A1/COL3A1 ratio. The higher ratio indicates a shift toward increased collagen type I production, which is essential for reinforcing the mechanical strength and stability of newly formed tissue. The significant fold change indicates that pddECM could promote more robust ECM remodeling, potentially fostering a collagen‐rich environment conducive to enhanced wound healing and tissue regeneration. Moreover, collagen synthesis and subsequent accumulation are associated with stabilization of the wound site and acceleration of wound healing.^[^ ^62^ ^]^ We confirmed collagen accumulation through picrosirius red staining, observing collagen deposition under our conditions, whereas no fibers were detected in the control group (Figure 5J).

### 2.6 Evaluation of Wound Healing, Vascularization, and Immunomodulatory Effects of the Bioengineered Skin Model

**Figure 6:**
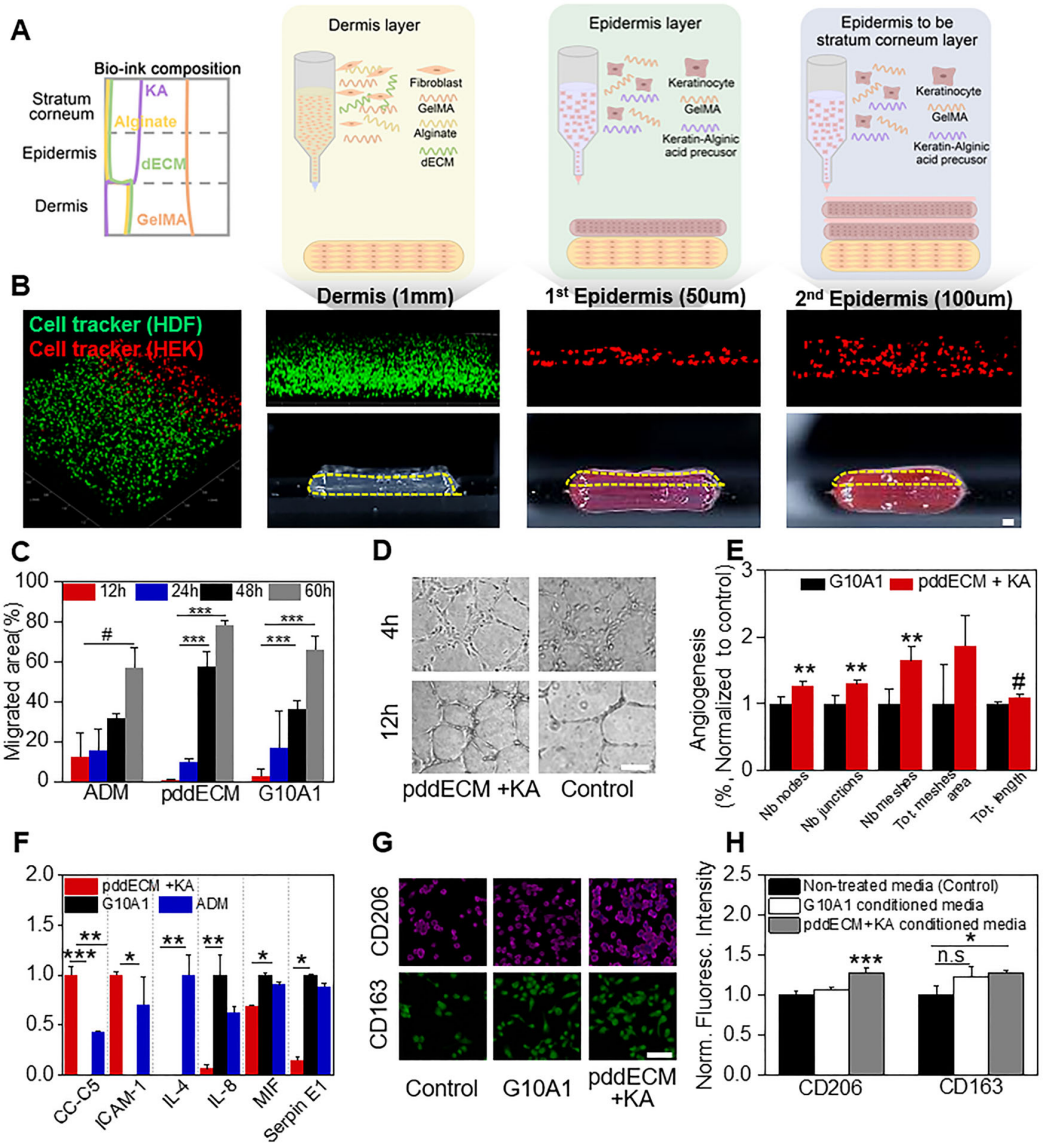
3D printed skin constructs: Functional validation in wound healing. A) Schematic of bioink composition according to layer the designed model structure. B) Bioprinted skin with CellTracker dye. Epidermis: keratinocyte (red) and fibroblsats (green). Scale bar = 1 mm. C) Analysis of migrated area among dermal layer bioink (pddECM), ADM and G10A1 (*n* = 3, ^#^ *p* < 0.01, ^***^ *p* < 0.0005). D) Optical images of angiogenesis for pddECM + KA and G10A1 at 4 and 12 h. Scale bar = 50 µm. E) Analyses of tube formation (total tube length, junction count, number of meshes, and mesh area) at 4 h for pddECM+KA and G10A1(*n* = 5, ^#^ *p* < 0.01, **p < 0.005, ns: no significance). F) Quantification of human cytokine expression under different conditions. All data were normalized to the condition with the lowest expression (*n* = 3, ^*^ *p* < 0.05, ^**^ *p* < 0.005, ^***^ *p* < 0.0005). G) Immunofluorescence images of M2‐specific markers (CD206 and CD163) in THP‐1–derived macrophages cultured under different conditions. Scale bar = 50 µm. H) Quantification of CD206 and CD163 fluorescence intensity from (G), normalized to PMA‐adhered cells without further stimulation (*n* = 4, ^*^ *p* < 0.05, ^***^ *p* < 0.0005).

The model was constructed with a dermal layer (GelMA 10%, alginate 1%, and pddECM 1%) and an epidermal layer (GelMA 10% and KA bioink 1%). The dermal layer was bioprinted as a single layer with a thickness of 1 mm, while the epidermal layer was printed twice, each with a thickness of 50 µm. To enhance adhesion between the epidermal layers, the printed single layer was coated with 1% gelatin (**Figure** **6**A). By employing a cell tracker, the structural integrity of the printed model was validated, and the spatial distribution and incorporation of cells within the layers were visualized (Figure 6B).

In skin graft procedures, Acellular Dermal Matrix (ADM) is implanted beneath the skin to promote tissue integration and accelerate wound healing. Accordingly, experiments were performed to assess wound closure (%) and α‐SMA expression in myofibroblasts, which are critical mediators of wound contraction, under the conditions of ADM, pddECM, and G10A1 (Figure 6C). At 60 h, wound closure rates for the ADM, pddECM, and G10A1 conditions were 56.81% (±10.34), 78.34% (±2.51), and 66.29% (±6.35), respectively. At 48 h, the pddECM condition exhibited a wound contraction of 57.92% (±7.45), demonstrating enhanced cell migration compared to the other conditions (1.81‐fold change relative to ADM at 48 h). ADM exhibits a modulus of 6.94 MPa with dense collagen fibers.^[^ ^63^, ^64^ ^]^ High‐stiffness matrices, like dense ECM, impede tissue integration and cell infiltration compared to low‐stiffness matrices. Accordingly, the superior fibroblast migration in the model is anticipated to enhance graft integration, cell infiltration, and wound healing.^[^ ^48^ ^]^

The microvasculature within damaged tissue is disrupted, leading to fluid accumulation, inflammation, and hypoxia.^[^ ^65^ ^]^ As oxygen supply is essential for hemostasis, collagen synthesis, and epithelialization, promoting vascularization is a key factor in wound healing.^[^ ^66^ ^]^ In this study, vascularization was evaluated by assessing HUVEC tube formation using conditioned media (Figure 6D & 6E). The model showed an increase in nodes, junctions, and meshes compared to G10A1, with ≈1.27, ≈1.30, and ≈1.64‐fold changes, respectively, at 4 h. These results reflect an expanded network due to neovascularization, suggesting the potential for enhanced vascularization and wound healing in skin graft applications. The immune response and intercellular signaling are critical to wound healing, with cytokine expression as a key indicator of its impact on tissue.^[^ ^67^, ^68^ ^]^ The cytokine array was performed under the experimental conditions of pddECM+KA, G10A1, and ADM (Figure 6F). Significant differences were identified in the expressions of complement component 5 (C5), ICAM‐1, IL‐4, IL‐8, macrophage migration inhibitory factor (MIF), and endothelial plasminogen activator inhibitor (Serpin E1). In our model, C5 expression increased ≈2.45‐fold compared to ADM, but was not detected under the G10A1 condition. C5 expression is known to accelerate wound healing, promote collagen deposition, and enhance hemostatic effects.^[^ ^69^ ^]^ ICAM‐1 expression was not detected in the G10A1 condition, but showed a 1.42‐fold increase in our model compared to ADM. ICAM‐1 contributes to wound healing by promoting keratinocyte migration to the wound center and facilitating tissue formation.^[^ ^70^ ^]^ In contrast, IL‐4 was not expressed in our model, and IL‐8, MIF, and Serpin E1 were expressed at low levels. The absence of IL‐4 may lead to insufficient macrophage activation and delayed collagen synthesis, although IL‐4 is also known to be a key driver of fibrosis.^[^ ^71^, ^72^ ^]^ Additionally, IL‐8 plays a role in wound closure and angiogenesis,^[^ ^73^ ^]^ but it can also contribute to inflammatory skin conditions and induce oxidative stress.^[^ ^74^, ^75^ ^]^ MIF activates genes involved in angiogenesis, re‐epithelialization, and wound contraction, but its overexpression can induce allergic and irritant contact dermatitis and may hinder wound healing.^[^ ^76^, ^77^ ^]^ Serpin E1 promotes fibroblast migration and induces differentiation into myofibroblasts, contributing to wound contraction. However, its overexpression may lead to fibrosis and tissue stiffening.^[^ ^78^ ^]^ Considering the interactions of these cytokines, the low expressions of IL‐4, IL‐8, and MIF suggest a potential delay in ECM remodeling and the inflammatory response. However, the elevated expressions of C5 and ICAM‐1 are likely to accelerate wound healing, promote collagen deposition, and facilitate keratinocyte migration, leading to effective tissue regeneration. Furthermore, the risk of complications such as fibrosis, inflammatory skin disorders, and tissue stiffening appears to be significantly reduced. This may allow tissue repair to proceed in a manner more closely resembling that of normal skin.

Proteomic analysis revealed that pddECM was enriched in proteins associated with extracellular matrix remodeling pathways (Figure 2). Collagen staining further demonstrated higher collagen synthesis and deposition in pddECM compared to G10A1 (Figure 5I). Based on these findings, we investigated whether pddECM+KA could modulate the polarization of M0 macrophages. THP‐1 cells were adherently cultured and treated for 48 h with either standard medium or conditioned media from each experimental group to analyze M1 and M2 marker expression (Figure 6G & 6H). B7‐1 (CD80) was used as the M1 marker, and CD206 and CD163 were used as M2 markers.^[^ ^79^, ^80^ ^]^ CD80 expression did not differ significantly among the conditions (Figure S10, Supporting Information). CD206 expression was slightly increased in the G10A1 and pddECM+KA conditions (≈1.06‐ and ≈1.27‐fold changes, respectively, relative to non‐treated THP‐1). CD163 expression was modestly increased in both the G10A1 and pddECM+KA conditions (≈1.23‐ and ≈1.27‐fold changes, in that order, compared to non‐treated THP‐1). This increase was not specific to the pddECM+KA condition and was also observed in G10A1. These results suggest that pddECM+KA may induce a CD206‐dominant M2‐like bias, particularly resembling an M2a‐like phenotype, rather than broadly promoting M2 polarization.

### 2.7 In Vivo Biocompatibility and Skin Regeneration Assessment of GelMA and pddECM‐Based Scaffolds

**Figure 7:**
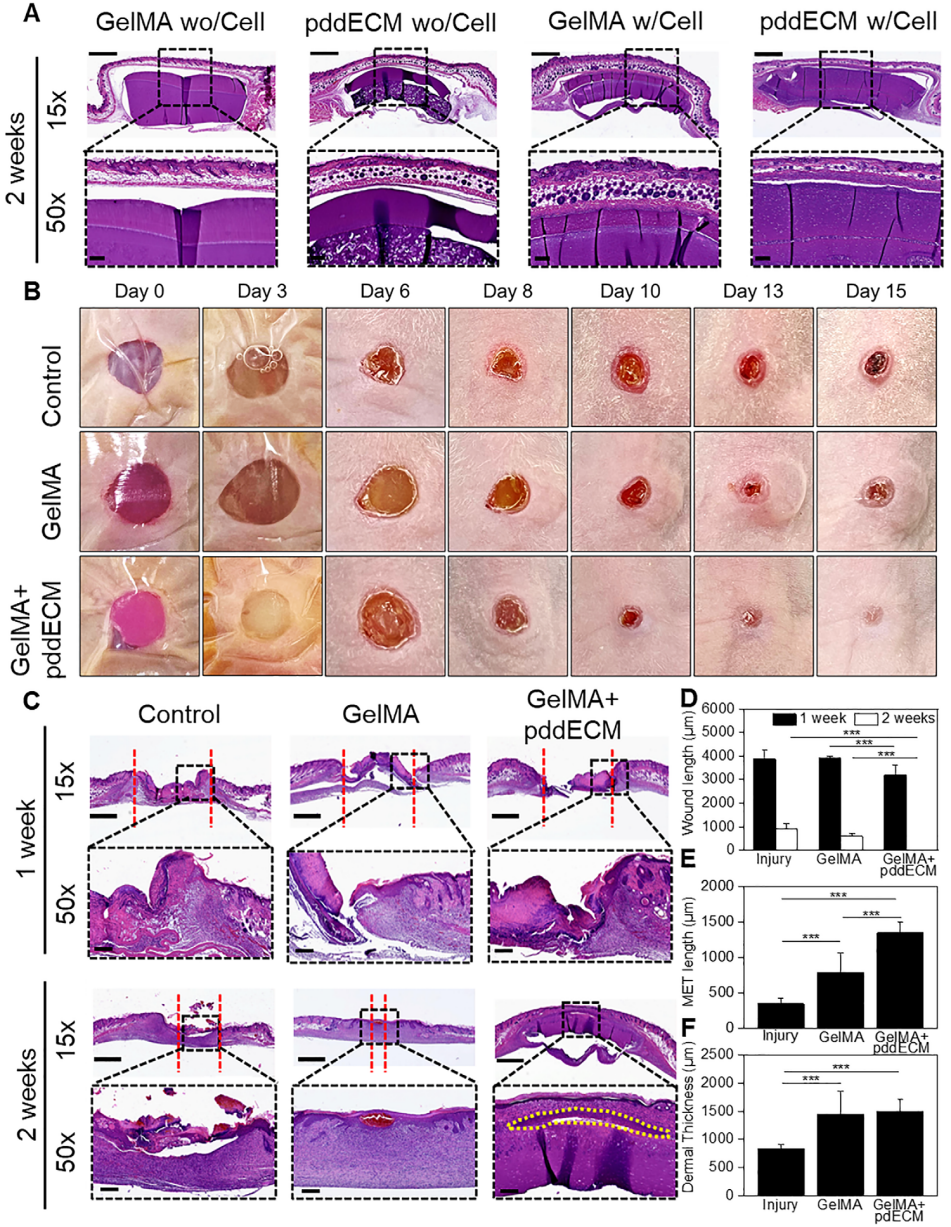
In vivo study. A) Histopathological results of subcutaneous implantation study. Scale bar for 15x = 1 mm, 50x = 200 µm. B) Gross evaluation of the wound healing study. C) Histopathological analysis of wound healing study. Scale bar for 15x = 1 mm, 50x = 200 µm. D) Quantitative analysis of wound length at 1 and 2 weeks (*n* = 10, ***p < 0.0005). E) Quantitative analysis of MET length at 1 week (*n* = 25, ***p < 0.0005). F) Quantitative analysis of dermal thickness at 1 week (*n* = 10, ***p < 0.0005).

To analyze the toxicity of GelMA and GelMA+pddECM scaffolds with or without cells, we used a mouse subcutaneous implantation model. For this purpose, we implanted the experimental materials (GelMA wo/Cell, GelMA+pddECM wo/Cell, GelMA w/Cell, GelMA+pddECM w/Cell) into the dorsal region of C57Bl/6 mice and performed a histopathological evaluation of local skin toxicity and vital organs (heart, lung, liver, kidney, spleen) for 2 weeks (**Figure** **7**A; Figure S11, Supporting Information). The results showed that, regardless of cell loading, the GelMA and pddECM groups did not induce significant inflammatory responses in the implanted subcutaneous area. However, the cell‐loaded groups (GelMA w/Cell, pddECM w/Cell) exhibited thicker fibrous capsules around the implanted material compared to the non‐cell‐loaded groups(GelMA wo/Cell, pddECM wo/Cell). In all tested groups, the experimental material implanted subcutaneously did not show any noticeable foreign body reaction in the surrounding tissue and exhibited a degradation pattern starting from the periphery. Histopathological evaluation of vital organs showed no significant toxic reactions in the heart, lungs, or kidneys (Figure S9, Supporting Information). However, mild to moderate reactive hepatocytes were observed in the liver, and mild proliferation of the splenic white pulp was also observed in the cell‐loaded groups(GelMA w/Cell, pddECM w/ECM). These results indicate that the transplantation of scaffolds and cells did not cause local or systemic toxicity in the transplanted animals.

To confirm whether the transplantation of GelMA and GelMA+pddECM‐based scaffolds enhances skin regeneration, a 6‐mm round‐shaped wound was made in nude mice, grafts were implanted, and analyzed their regenerative properties for 2 weeks. To prevent the grafts from falling off the wound site, 3 M Tegaderm was applied for 3 days. As observed in Figure 7B, gross examination revealed that there was no significant change in wound size until day 7, but from day 13, improved wound healing was observed in the scaffold implantation groups (GelMA, GelMA+pddECM). In particular, skin regeneration was complete in the GelMA+pddECM group by day 15, but wound crust remained in the control group, indicating that regeneration was not complete.

In the histopathological evaluation, none of the groups had achieved complete skin regeneration by the first week, and inflammation was observed at the wound site. In the control group, incomplete migration of the epidermal tongue was observed along with prominent inflammation, whereas in the GelMA+pddECM group, the length of the epidermal tongue increased. At week 2, the epidermis was not completely regenerated in the control group, and discontinuous epidermis in the form of crusts was observed, whereas in the GelMA+pddECM group, the epidermis was completely regenerated in all animals. In the GelMA group, part of incomplete epidermis and congestion were observed in the epidermal area. Interestingly, as shown by the yellow dotted line in Figure 7C, the GelMA+pddECM implant exhibited epidermal regeneration resembling the stratum basale layer (Figure 7C, yellow line).

Quantification results showed a statistically significant difference in wound length between the GelMA+pddECM group, which demonstrated complete healing by week 2, and the remaining groups (Figure 7D). In particular, the MET length was ≈2.3 times longer in the GelMA group and ≈3.9 times longer in the GelMA+pddECM group compared to the control group. Considering that the GelMA+pddECM group showed a MET length that was 1.7 times longer than that of the GelMA group, the addition of pddECM can be seen to enhance epidermal regeneration (Figure 7E). Regarding dermal thickness at week 1, both the GelMA and GelMA+pddECM groups showed a significant increase compared to the control group, and there was no notable difference between the GelMA and GelMA+pddECM groups (Figure 7F).

## 3 Conclusion

Our study demonstrates that the pddECM and KA‐based bioink successfully replicates key structural and functional attributes of native skin in a patient‐specific skin graft model. The expression of basal (KRT14) and differentiation markers (KRT10, FLG) indicated maintenance of normal epidermal differentiation and keratinization without premature desquamation. Enhanced collagen type I synthesis and accumulation within the dermal layer supported robust ECM production and remodeling. In vivo, the model promoted neovascularization and, unlike the control, achieved complete wound closure by day 15 with a marked increase in epidermal tongue formation. While dECM retains the intrinsic biochemical complexity and microarchitecture of native tissue, it is limited by low mechanical strength and complex preparation. To address these issues, we employed a hybrid strategy combining GelMA and alginate to reinforce structural stability. Unlike conventional reinforcement strategies that rely on non‐human dECM sources such as porcine or fish tissues, our approach utilized patient‐derived dECM to achieve precise immunological compatibility and functional personalization. This is particularly advantageous for conditions such as diabetic foot ulcers and chronic inflammatory wounds, where patient‐derived dECM may reduce the risk of immune rejection and graft failure. Recognizing the limited availability of donor tissue, we plan to explore autologous adipose tissue‐derived dECM as a more accessible and stable source for future applications.

## 4 Experimental Section

### Ethical Approval, Tissue Collection, and Primary Cell Isolation

This study was approved by the Institutional Review Board of Ewha Womans University Hospital (approval number: SEUMC 2022‐10‐065‐018), and written informed consent was obtained from all participants in accordance with the approved protocol. All procedures were conducted in compliance with institutional and national ethical guidelines.

Skin tissues were obtained from the donor sites (leg and groin) during lower extremity reconstructive surgeries in patients aged 60–70 years. Only tissues with no visible signs of fibrosis, inflammation, or necrosis were used, and any areas damaged by electrocautery were completely excised prior to use. The tissues were wrapped in saline‐soaked gauze and stored at 4 °C. Before tissue dissociation, blades and forceps were sterilized and disinfected with ethanol. Cells were isolated using dispase II (Gibco, 17105041), collagenase I (Gibco, 17100‐017), 0.25% trypsin‐EDTA (Gibco, 25200056), and a cell strainer (SPL Life Sciences, 93070). After a 3‐day stabilization period, the tissue was removed, and the culture medium was replaced. Fibroblasts were cultured in high‐glucose Dulbecco's Modified Eagle Medium (DMEM) (Gibco, 11965092) supplemented with FBS (Corning, 35‐010‐CF), and keratinocytes were cultured in KGM (Lonza, 00192060). All media were supplemented with Penicillin‐streptomycin (Gibco, 15140) and amphotericin B (Sigma–Aldrich, A2942).

### Decellularization and Processing of pddECM

The decellularization process of the dermis was conducted as follows: 1) washing and mincing the tissue; 2) treatment with 0.025% trypsin‐EDTA for 14 h; 3) EDTA‐trypsin treatment; 4) DNase treatment; 5) disinfection with 0.1% peracetic acid and ethanol. Between each step, the tissue was washed with PBS and triple‐distilled water for 24–48 h. The decellularized tissue was stored at −20 °C for 1 day, followed by freeze‐drying for at least 2 days. The tissue was then pulverized using a FM‐200 grinder, and digestion was performed with pepsin at 10% of the dECM weight. The dECM pre‐hydrogel concentration was set to 5% and processed in 0.05 M HCl.

### Assessment of Decellularization Efficiency and ECM Component Retention

To confirm the success of decellularization, double‐stranded DNA (dsDNA), GAGs, and collagen were quantified. These analyses confirmed the removal of immunogenic components and the preservation of ECM components. Freeze‐dried native tissue and pddECM samples were minced prior to analysis. For dsDNA quantification, the GeneJET Genomic DNA Purification Kit (#K0702, Thermo Fisher Scientific, USA) was used, and measurements were taken with a Quant‐iT PicoGreen dsDNA Assay Kit (#P11496, Invitrogen, USA). GAGs and collagen were digested with papain solution at 60 °C for 16 h. The papain solution was prepared with 25 mg mL^−1^ papain from Carica papaya (76216‐50MG), Na2‐EDTA (03690‐100ML), sodium phosphate dibasic (Na_2_HPO_4_) (795410‐100G), sodium phosphate monobasic (NaH2PO4) (S2554‐100G), and cysteine HCl (C121800‐5G) at pH 6.6. GAGs and collagen were quantified using the Sulfated Glycosaminoglycan Quantification Kit (AMSbio, 280560‐N) and the Picosens Hydroxyproline Assay Kit (Biomax, BM‐HYP‐100), respectively. All measurements were performed on a microplate reader, according to the manufacturers’ instructions.

### Tissue Processing and Staining for Structural Characterization

Native dermis tissue and pddECM samples were prepared by freezing and freeze‐drying. The samples were fixed in 4% paraformaldehyde for 1 h per 1 mm of thickness. They were then dehydrated by varying concentrations of EtOH and sucrose. After freezing at 4 °C for 1 day, the samples were embedded in OCT molds and sectioned at 20 µm thickness. Hematoxylin and Eosin staining (Abcam, ab245880) and picrosirius red staining (Abcam, ab246832) were performed on the samples according to the manufacturer's protocols. Immunofluorescence staining for DAPI, COL1A1 (Abclonal, A22090), and COL3A1 (Santa, sc‐271249) was also conducted. After staining, 1–2 drops of mounting solution were applied to each sample, and the samples were covered with a cover glass to seal.

### Sample Preparation for Scanning Electron Microscopy (SEM)

The pddECM and native dermis tissue samples were prepared as follows: 1) fixation in 4% PFA for 1 h per 1 mm thickness at 25 °C, 2) washing three times with PBS, 30 min each, and 3) washing with deionized water for 30 min. The samples were then dehydrated by gradually increasing ethanol concentrations: 1) 50% ethanol, twice for 10 min, 2) 70% ethanol, twice for 10 min, 3) 95% ethanol, twice for 10 min, and 4) 100% ethanol, three times for 15 min. Dehydration was further carried out with hexamethyldisilazane (HMDS, #440191, Sigma–Aldrich) using the following ratios of HMDS to ethanol: 1) 1:2 for 15 min, 2) 1:1 for 15 min, and 3) 2:1 for 14 min. Finally, the samples were treated with 100% HMDS three times for 15 min each and left submerged in HMDS in a fume hood overnight until complete evaporation.

### Proteomic Sample Preparation and LC‐MS/MS Analysis

The pddECM and tissue samples were processed using the Bravo FASP (Filter‐Aided Sample Preparation) digestion protocol. Each sample was loaded into a filter plate under vacuum, and digestion was performed using Agilent's Bravo automated liquid handling system. Protein reduction was achieved by adding 500 mM TCEP (final concentration: 5 mM), followed by shaking at 300 rpm for 1 min at 33 °C, and then incubating for 30 min. Alkylation of proteins was subsequently carried out by adding 500 mM IAA (final concentration: 50 mM), shaking at 500 rpm for 1 min at 25 °C, and incubating for 1 h in the dark. Proteolytic digestion was performed with trypsin at a 1:50 enzyme‐to‐protein ratio, followed by overnight incubation at 37 °C. Post‐digestion, peptides were desalted using a C18 Micro Spin‐Column, concentrated using a Speed‐vac, and stored at −20 °C until further analysis. For LC‐MS/MS analysis, peptides were separated using C18 columns. The trapping column was a C18 3 µm 100 Å, 75 µm × 2 cm, and the analytical column was a PepMap RSLC C18 2 µm 100 Å, 75 µm × 50 cm. The mobile phases consisted of A: water containing 0.1% formic acid, and B: 80% acetonitrile with 0.1% formic acid. A gradient elution was applied, increasing solvent B from 4% to 96% over a total of 185 min, with a flow rate of 300 nL min^−1^. Proteomic data were analyzed using Proteome Discoverer software, with the Uniprot Homo sapiens database utilized for protein identification. Carbamidomethylation of cysteine residues was set as a fixed modification, while variable modifications included methionine oxidation, protein N‐terminal acetylation, and carbamylation.

### Preparation and Optimization of Dermal and Epidermal Bioinks

GelMA was synthesized using porcine gelatin (G1890‐1KG, Sigma–Aldrich) and methacrylic anhydride (276685‐500 mL, Sigma–Aldrich). For the dermal ink, sodium alginate (W201502, Sigma–Aldrich) and decellularized matrix derived from patient tissue were used. The optimized concentrations were 10% GelMA, 1% alginate, and 1% pddECM. For the epidermal layer, the ink was optimized with 10% GelMA and 1% KA bioink (Biofriends). All inks contained 0.5% photo‐initiator. The initial concentrations of GelMA, alginate, and KA, accounting for dilution, were maintained at 80 °C for 10 min in PBS containing the photo‐initiator. The pddECM was then added and mixed at 37 °C.

### Rheological and Mechanical Characterization of Bioinks

All rheological analyses were conducted using a rheometer (Discovery HR‐2, TA Instruments, USA) with a 20 mm diameter parallel‐plate geometry (TA Instruments, 115796) at a 200 µm gap. The viscosity was measured with a shear rate sweep ranging from 0.1 to 100 s^−1^ at 25 °C. Additionally, the viscosity changes were measured between 4 and 37 °C. The complex modulus was assessed using an oscillation amplitude‐logarithmic sweep from 1.0% to 100% strain at 1.0 Hz, performed at 25 °C. The compressive strength was measured at a speed of 33.33 µm s^−1^, with samples prepared at a diameter of 8 mm and a thickness of 1 mm.

### Swelling and Degradation Analysis of Hydrogel Constructs

The swelling/degradation ratio was analyzed by comparing the initial weight of the samples with their weight changes over time. Weights were measured at 0, 1, 3, 6, 12 h, and on days 1, 4, 7, 14, 21, and 28. The samples were immersed in DMEM media and incubated at 37 °C.

### Immunofluorescence Staining of Hydrogel Samples

Hydrogel were washed with PBS for 30 min 2 times and fixed in 4% paraformaldehyde for 1 h. The samples were permeabilized in 0.1% Triton X‐100 in PBS and blocked with 1% bovine serum albumin (BSA), both for 1 h at RT. After the initial blocking step, samples were incubated overnight with primary antibodies at 4 °C and washed twice with PBS. Next, they were blocked again in PBS with 1% BSA and 2% goat serum for 1 h at room temperature, followed by a 40‐min incubation with secondary antibodies at 37 °C. Finally, the samples were washed twice with PBS and analyzed using a confocal microscope.

### In Vitro Wound Healing Assay for HDF Migration

To assess HDF migration, bioink was thinly coated on a 24‐well plate and UV cross‐linking was performed. HDFs were seeded at 75 000 cells cm^−^^2^ and allowed to stabilize for 48 h. Wounds were created using a pipette tip, and migration was monitored at various time points.

### In Vitro Angiogenesis Assay with HUVECs

The tube formation assay was conducted using HUVECs and Geltrex (Gibco, A1413202) to evaluate the cells’ capacity for vascular structure formation. Each well of a 24‐well plate was loaded with 150 µL of Geltrex and incubated at 37 °C for 30 min to allow for gelation. HUVECs were plated at a density of 9 × 10⁴ cells per well in Geltrex‐coated wells, using 0.5 mL of either conditioned medium or control medium. Images were obtained at 4, 8, and 12 h intervals, and each time point was captured using fluorescence microscopy. Various characteristics of capillary‐like tube formation, such as total tube length, junction count, number of meshes, and mesh area, were analyzed using the “Angiogenesis Analyzer” plugin for ImageJ software (Carpentier, G. Angiogenesis Analyzer for ImageJ. ImageJ News, 9 November 2012).

### Cytokine Profiling via Human Cytokine Array

The cytokines associated were assessed utilizing the Proteome Profiler Human Cytokine Array Kit (R&D Systems, ARY005B) by the manufacturer's instructions. For the experiment, cell culture mediums of each sample were collected at 14 days and stored immediately at −80 °C. Analysis images were obtained using the Amersham ImageQuant 800 (Cytiva) system.

### Animal Models for Biocompatibility and Regenerative Efficacy

In vivo experiments were conducted using murine models of subcutaneous implantation and epidermal wound healing. All animal study was performed in accordance with the guidelines of the IACUC of Korea University (Approval Number: KUIACUC‐2024‐0088). For the subcutaneous implantation study, twelve C57Bl/6 mice (male, 7 weeks) were used and categorized into four groups based on the composition of the constructs. The constructs consisted of a dermal layer composed of 10% GelMA, 1% alginate, and 1% pddECM, and an epidermal layer containing 10% GelMA and 1% KA bioink. Antibiotics (enrofloxacin, 5 mg kg^−1^) and analgesics(carprofen, 5 mg kg^−1^) were injected to the animal before surgery. Inhalation anesthesia was induced using 4% isoflurane mixed with oxygen, and anesthesia was maintained with 1.5–2.5% isoflurane. For subcutaneous implantation, the implantation site (dorsal skin) was disinfected with povidone/70% alcohol, and then incised with Metzenbaum scissors to a length of ≈1 cm. The incised skin was bluntly dissected with blunt scissors, and a 6 mm diameter circular sample was transplanted. The animals were sacrificed at 2 weeks post‐transplantation, with three animals per group. On the day of sample collection, animals were euthanized using CO_2_, and skin and vital organs (heart, lung, liver, kidney, spleen) were harvested and fixed in 4% neutralized buffered formalin.

For the wound healing model, eighteen nude mice (male, 7 weeks) were used and categorized into three groups. The samples included a control group with 10% GelMA alone and an experimental group incorporating 10% GelMA with 1% pddECM. Premedication and anesthesia were administered in the same manner as in the subcutaneous study. A 6 mm circular wound was created using a 6 mm biopsy punch, and a 6 mm scaffold was implanted into the wound in the scaffold implantation group. After implantation, 3 M Tegaderm was used to prevent the implant from detaching from the wound. The implanted animals were euthanized 1 and 2 weeks after implantation, and the skin was collected and fixed in 10% NBF.

### Histopathological Processing and Analysis

Formalin‐fixed tissue samples were processed using standard paraffin tissue processing methods (dehydration and paraffin infiltration). Paraffin block samples were sectioned into 4–5 µm thick slides using a microtome, followed by deparaffinization/rehydration and hematoxylin and eosin (HE) staining. The stained slides were fixed in mounting medium and scanned using a histopathological slide scanner (Motic microscope, MoticEasyScan One, USA), and images were obtained and analyzed using QuPath software.

### Statistical Analysis

All data are presented as mean ± standard deviation (SD). Error bars indicate the SD. Two‐group comparisons were analyzed using a two‐tailed unpaired Student's *t*‐test. One‐way ANOVA was used for comparisons among multiple groups. Statistical significance was defined as *p < 0.05. *#p* < 0.01; **p < 0.005; ***p < 0.0005. All experiments were independently performed at least three times. All data were analyzed using ANOVA, and in cases with more than three groups, pairwise comparisons were performed to account for potential errors arising from multiple comparisons.

## Conflict of Interest

The authors declare no conflict of interest.

## Supporting information



Supporting Information

## Data Availability

The data that support the findings of this study are available from the corresponding author upon reasonable request.
