# Supplementary material for: Bioengineered Skin Grafts from Patient‐Derived Decellularized Extracellular Matrix and Autologous Cells for Personalized Regenerative
Source: Adv Sci (Weinh). 2025 Sep 25;12(44):e11889. doi: 10.1002/advs.202511889 (PMC12667513; doi:10.1002/advs.202511889)
Supplement: Supplementary file 1 — Supporting Information [file ADVS-12-e11889-s001.docx]

Supporting Information

**Bioengineered Skin Grafts from Patient-Derived Decellularized Extracellular**

**Matrix and Autologous Cells for Personalized Regenerative**

*Raehui Kang, Suyeon Shin,* *Yurim Choi, WonJun Jang, Soojin Park, Mi Kyung Lee, Hyun-Jong Cho, Yu Shrike Zhang, Han-Jun Kim^*^, Bo Young Park^*^, Junmin Lee^*^*

**Figure S1.** Quantification data of hydroxyproline content in dermis-derived decellularized extracellular matrix (dECM) and native dermis tissue. Hydroxyproline content indicating collagen presence (n=3, ns: no significance).

**Figure S2**. Quantification data of DNA content in dermis-derived decellularized extracellular matrix (dECM) and native dermis tissue. Hydroxyproline content indicating collagen presence. Data are presented as mean ± standard deviation (n=3, ***p < 0.0005).


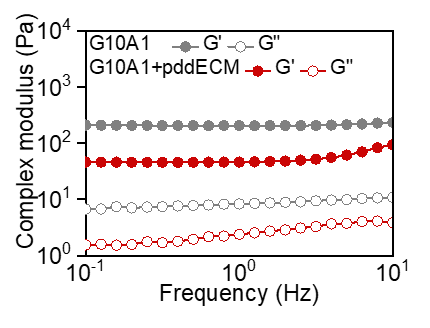


**Figure S3**. Frequency sweep data of complex moduli (G′ and G″) for G10A1 and G10A1 + pddECM bioinks over the range of 0.1–10 Hz. Data are presented as mean ± standard deviation.


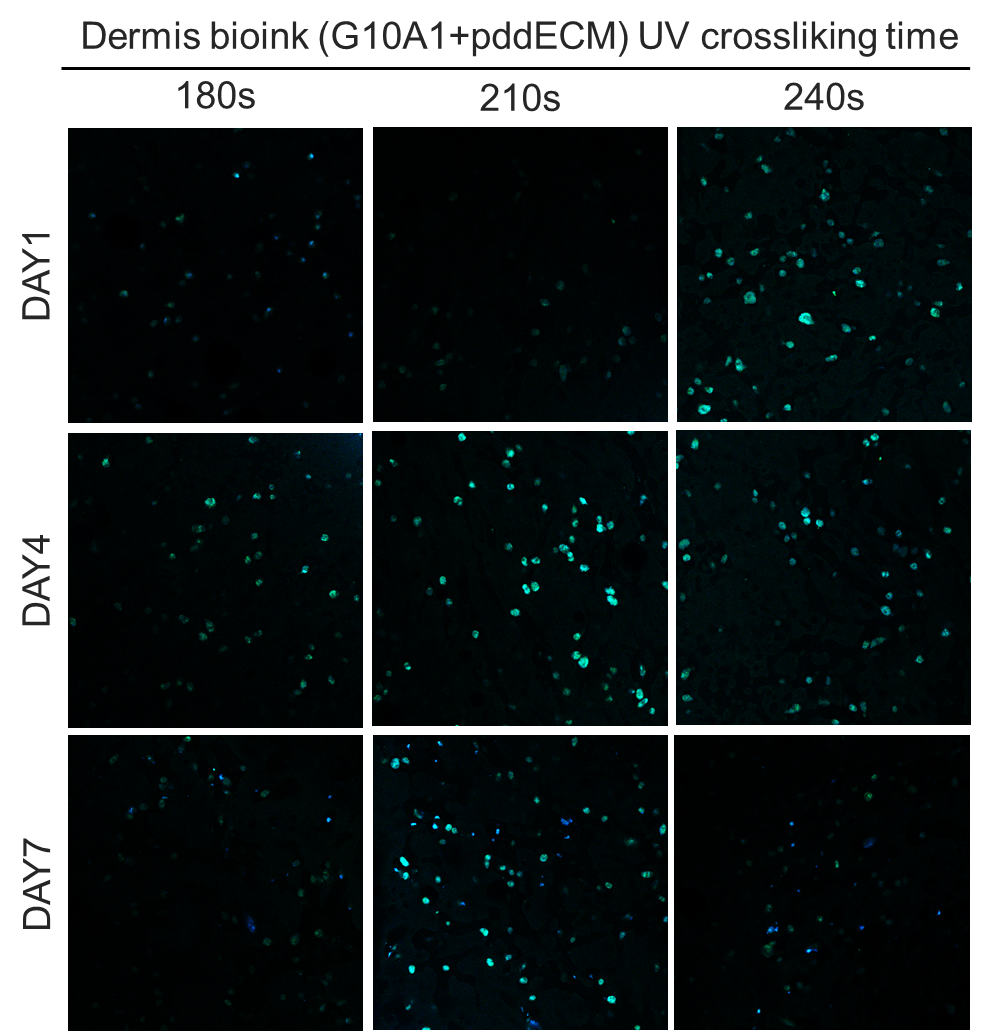


**Figure S4**. Representative immunofluorescence images showing Ki-67 expression in the dermal layer of the implanted constructs. Cell nuclei were counterstained with DAPI (blue). Scale bar = 100 µm.


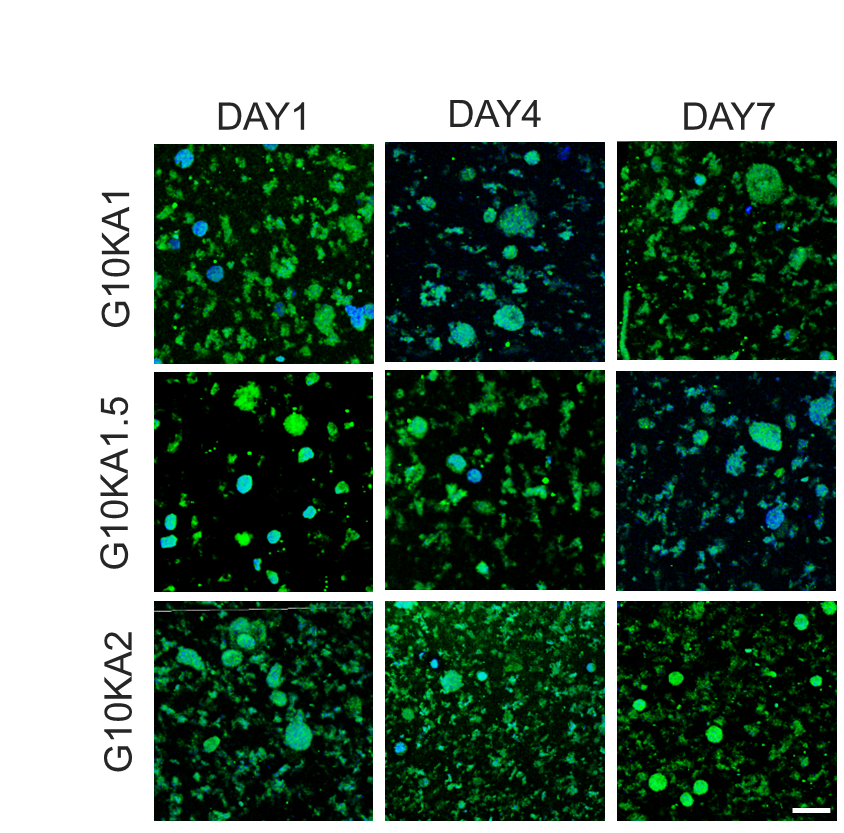


**Figure S5**. Representative immunofluorescence images showing Ki-67 expression in the epidermal layer of the implanted constructs. Cell nuclei were counterstained with DAPI (blue). Scale bar = 100 µm.


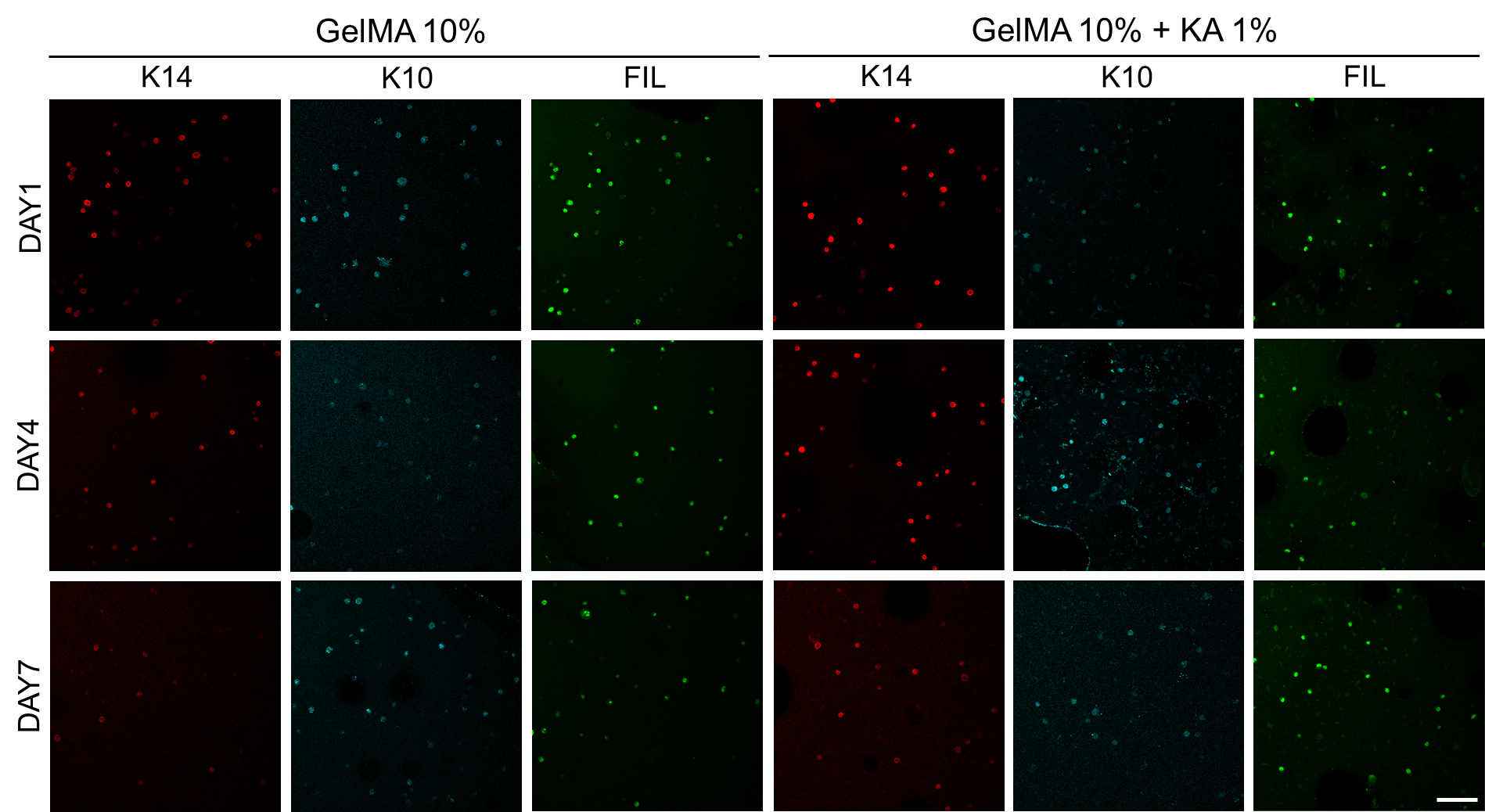


Figure S6. Images of immunofluorescence intensity for the expressions of markers K10, K14, and filaggrin, assessing epidermal function markers with and without KA ink . Scale bar = 100 µm.


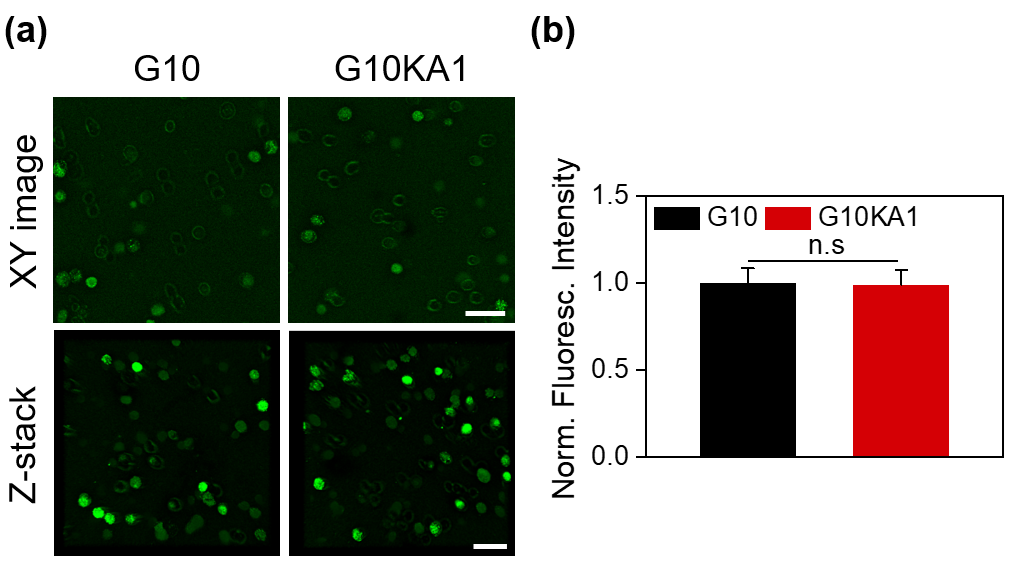


Figure S7. Immunofluorescence images and quantification of ZO-1 expression in G10 and G10KA1 hydrogels on Day 14. (a) Representative XY-plane and Z-stack images of ZO-1 staining (green). Scale bar = 50 µm. (b) Quantification of normalized fluorescence intensity showing no significant difference between groups (n=4, ns: no significance).

Figure S8. Quantification of normalized transepithelial electrical resistance (TEER) for G10 and G10KA1 hydrogels on Days 1 and 14 (n=4, *p < 0.05, ^#^p < 0.01).


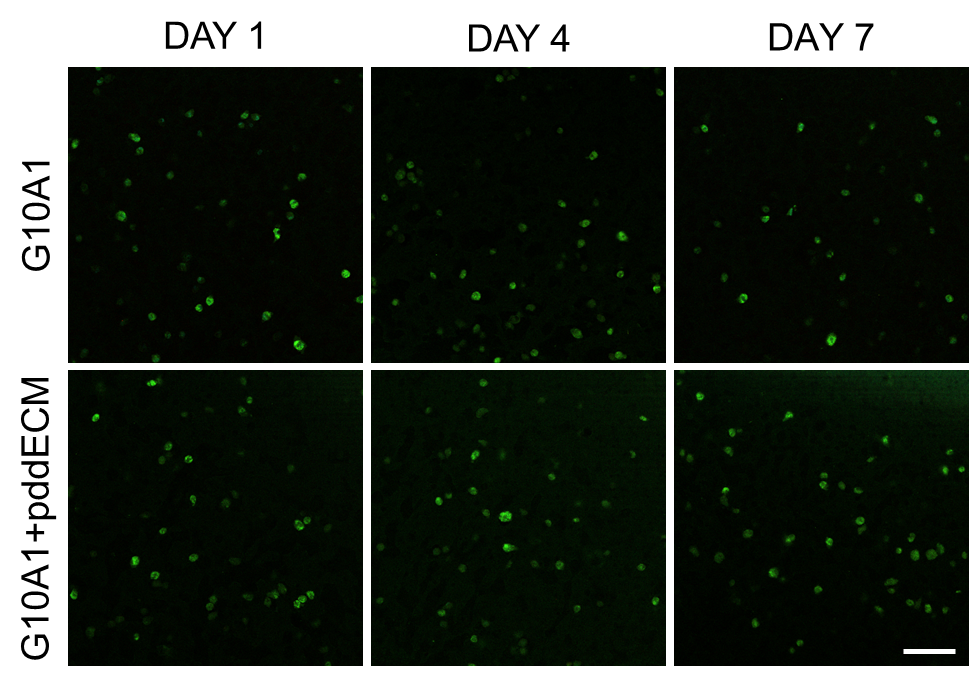


Figure S9. Representative immunofluorescence images showing collagen 1 alpha 1 (COL1A1) staining (green) in cell-laden G10A1+pddECM hydrogels. Scale bar = 100 µm.


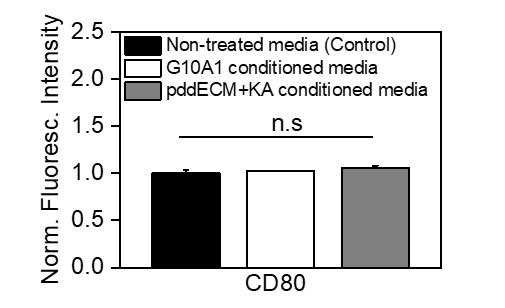


Figure S10. Quantification of CD80 fluorescence intensity normalized to PMA-adhered cells without further stimulation (n=4, ns: no significance).


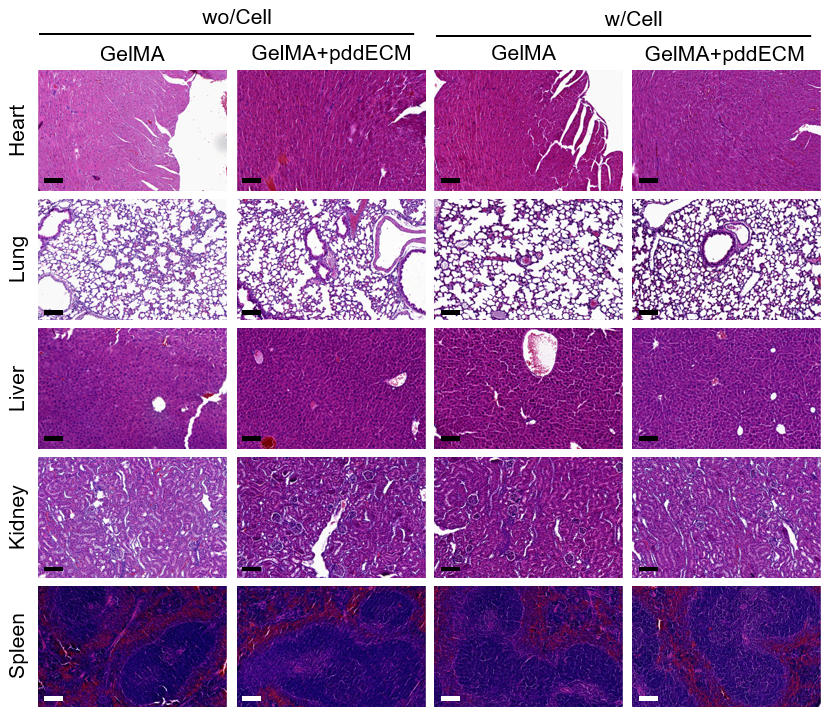


**Figure S11.** H&E-stained images of major organs after implantation of GelMA and GelMA + pddECM hydrogels for histological toxicity evaluation. Scale bar = 100 µm.
